# Comprehensive analysis of ZFPM2-AS1 prognostic value, immune microenvironment, drug sensitivity, and co-expression network: from gastric adenocarcinoma to pan-cancers

**DOI:** 10.1007/s12672-022-00487-0

**Published:** 2022-04-13

**Authors:** Di Chen, Mengmeng Wang, Xin Jiang, Zhifan Xiong

**Affiliations:** Department of Gastroenterology, Liyuan Hospital, Tongji Medical College, Huazhong University of Science and Technology, Wuhan, 430061 China

**Keywords:** Pan-cancers, Tumor microenvironment, ZFPM2-AS1, Infiltrating immune cells, Drug sensitivity, Molecular subtype

## Abstract

**Background:**

ZFPM2-AS1, as an oncogenic lncRNA, plays an essential role in the progression of several tumors. However, the prognostic significance, biological function, and molecular mechanism of ZFPM2-AS1 in most tumors have not been fully elucidated.

**Methods:**

We analyzed differentially expressed immune-related lncRNAs (IRlncRNAs) and clustered gastric adenocarcinoma (GAC) samples based on these lncRNAs expression. Then, WGCNA and survival analysis were performed to determine key IRlncRNA (ZFPM2-AS1) in GAC. The comprehensive analysis was performed to evaluate the association between ZFPM2-AS1 expression and survival, tumor microenvironment (TME), immune-related factors, and related signal pathways in pan-cancers. Furthermore, we constructed a co-expression network of ZFPM2-AS1, and NUP107 and C8orf76 were identified as target mRNAs. We further evaluated the role of NUP107 and C8orf76 in the GAC microenvironment. More importantly, real-time polymerase chain reaction (qRT-PCR) was employed to validate ZFPM2-AS1, NUP107 and C8orf76 expression.

**Results:**

ZFPM2-AS1 was remarkably overexpressed and correlated with poor overall survival in most tumors. Further analysis showed that ZFPM2-AS1 was related to various immune cells infiltrated in the microenvironment of most tumors. GSEA revealed that ZFPM2-AS1 in GAC was primarily involved in immune-related pathways. Furthermore, NUP107 and C8orf76 were identified as potential target mRNAs of ZFPM2-AS1, which was related to infiltrating immune cells in the GAC microenvironment. qRT-PCR verified that ZFPM2-AS, NUP107 and C8orf76 were highly expressed in gastric cancer cells.

**Conclusion:**

ZFPM2-AS1 could be a potential biomarker for cancer prognosis, and a promising immune target for cancer therapy. Furthermore, ZFPM2-AS1 might play an immunosuppressive role in the GAC microenvironment.

**Supplementary Information:**

The online version contains supplementary material available at 10.1007/s12672-022-00487-0.

## Introduction

Long non-coding RNAs (lncRNAs) are a heterogeneous class of RNA molecules with longer than 200 nucleotides, which locate within inter-genic region in the genome [1, 2]. LncRNAs are numerous and diverse; according to most estimates, the number of human lncRNA is much higher than that of protein-coding genes [3]. Based on the subcellular localization, lncRNAs can be divided into cytoplasmic lncRNAs and nuclear lncRNAs. Cytoplasmic lncRNAs play a crucial role in post-transcriptional regulation, such as crosstalking with mRNAs via sponging miRNAs. Nuclear lncRNAs mainly participate in the modulation of epigenetic events, transcription or mRNA processing [4]. With the advancement of RNA sequencing technologies and computational prediction techniques, the total number of lncRNAs is continuously increasing [5, 6]. Their growing ranks have attracted the attention of the cancer research community. In the past few decades, accumulating evidence has elucidated the crucial function of lncRNAs in modulating various biological processes of cancer, such as differentiation, apoptosis, DNA damage regulation, and immune response [7–9]. LncRNA ZFPM2-AS1, located on chromosome 8q23, has been reported to participate in the occurrence and development of several cancers. For example, ZFPM2-AS1 contributes to lung adenocarcinoma cell growth and epithelial to mesenchymal transition [10]. ZFPM2-AS1 also facilitates hepatocellular carcinoma (HCC) cell cycle progression, cell proliferation, and invasion via miR-653/GOLM1 [11]. In addition, ZFPM2-AS1 promotes the growth, migration, and invasion of thyroid cancer cells through the miR-515-5p/TUSC3 axis [12]. However, the expression pattern, biological function, molecular mechanism, and prognostic significance of ZFPM2-AS1 in most tumor types have not been fully elucidated.

The tumor microenvironment (TME) refers to the physiological and biochemical elements around tumor cells, including the extracellular matrix, the tumor vascular system, stromal cells, immune cells, and the acidic and hypoxic environment [13–15]. A growing body of studies has suggested that TME has profound impacts on tumor development and therapeutic responses. For instance, differences of immune cells infiltrated in the TME, including tumor-associated macrophages (TAMs), dendritic cells, and T lymphocytes, have been found in tumor patients [16, 17]. Furthermore, CD8^+^ T cells, TAMs, and CD4^+^ T cells in the TME relate to clinical outcomes of multiply tumors, including lung cancer, urothelial cancer, melanoma, and gastric cancer [18–20]. Recently, lncRNAs have been studied for their promising role in the regulation of TME. LncRNAs contribute to controlling and mediating the interaction of tumor cells and infiltrating immune cells in the TME, as well as essential mechanisms of immune response [21]. Therefore, the correlation between ZFPM2-AS1 and immune cells infiltrated in the TME is helpful to understand the function of ZFPM2-AS1 in the development of tumors.

Chemotherapy is the first-line therapeutic plan for advanced GC patients, which can significantly improve their life quality and overall survival rate [4]. The widely used chemotherapeutic drugs primarily include platinum drugs, 5-fluorouracil (5-FU), epirubicin, docetaxel, and so on [22]. However, the development of drug resistance becomes a major challenge in clinic oncology, resulting in tumor recurrence and metastasis. Although the detailed mechanism of drug resistance is still inconclusive, lncRNAs have received increased attention to improving the effectiveness of anticancer therapy [23]. Recently, accumulating studies have revealed that lncRNAs are involved in the chemoresistance of GC via various mechanisms, including the induction of autophagy, inhibition of apoptosis, modulation of drug resistance-related genes and signaling pathways, and regulation of cancer stem cells [24]. Therefore, exploring the effect of ZFPM2-AS1 on drug resistance is helpful to enhance therapy efficiency of GC patients. In this study, the non-negative matrix factorization (NMF) algorithm was applied to cluster gastric adenocarcinoma (GAC) samples, and Kaplan–Meier analysis, ESTIMATE, and CIBERSORT algorithms were used to estimate the survival and immune microenvironment differences between clusters. Then, a key immune-related lncRNA (ZFPM2-AS1) was identified associated with clusters and prognosis of GAC patients via Weighted Gene Co-Expression Network Analysis (WGCNA) and survival analysis. We further analyzed the differential expression, clinical prognosis, TME, tumor mutational burden (TMB), microsatellite instability status (MSI), drug sensitivity, and related signal pathways of ZFPM2-AS1 in 33 tumor types. Furthermore, we further constructed a ZFPM2-AS1-mRNA co-expression network and performed differential expression, survival, TMB, MSI, TME, and immune subtype analysis of target mRNAs (NUP107 and C8orf76). Finally, several experiments were carried out to verify the results of bioinformatics analysis. Our study provides a theoretical basis for therapeutic targets and novel mechanisms of ZFPM2-AS1 in GAC and pan-cancers.

## Materials and methods

### Data collection and differentially expressed immune-related lncRNAs (DE-IRlncRNAs) analysis

**Fig. 1:**
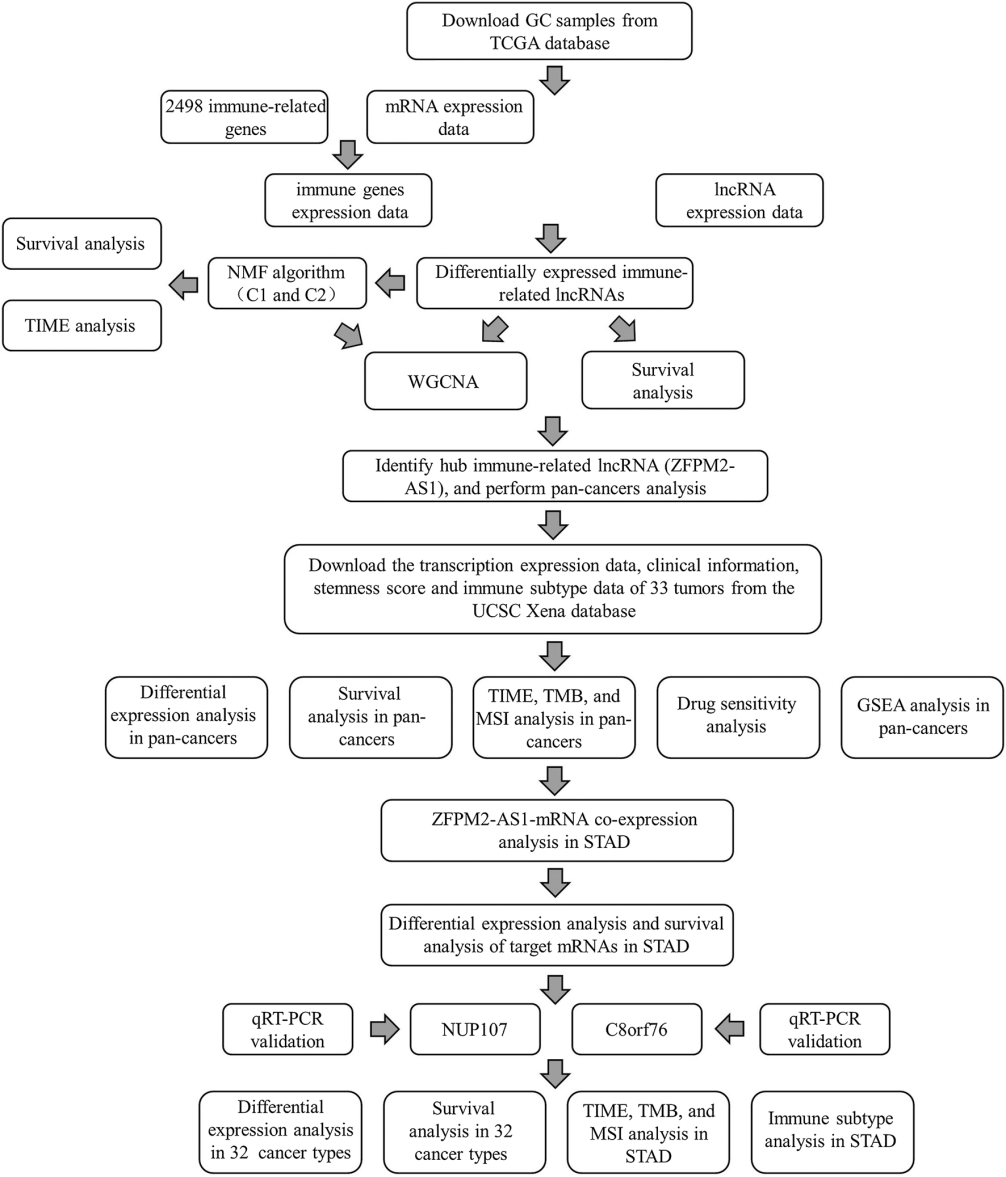
Flowchart of the pan-cancer analysis of ZFPM2-AS1

The flow diagram of the study is shown in Fig. 1. The lncRNA data and clinical information of 339 GAC and 30 normal samples were extracted from the TCGA. Subsequently, the list of 2498 immune-related genes (IRGs) was obtained from the ImmPort database. To identify the relationship between IRGs and corresponding lncRNAs expresion, Pearson correlation analysis was carried out in the R software. The immune-related lncRNAs (IRlncRNAs) were determined according to the correlation coefficient (|R| > 0.4 and *P* < 0.001). Furthermore, we identified differentially expressed lncRNAs (DElncRNAs) in GAC samples using the R package “limma”. |Log2FC| > 1 and FDR < 0.05 were regarded as the threshold. Finally, the overlapping lncRNAs between IRlncRNAs and DElncRNAs were determined as DE-IRlncRNAs for further analysis.

### Identification of molecular subtypes and assessment of immune microenvironment

The NMF algorithm is a powerful technique for identifying distinct molecular subtypes and evaluating the optimal number of clusters based on gene expression microarrays. According to the expression of DE-IRlncRNAs, 339 GAC samples were clustered by the NMF algorithm. The optimal number of clusters and the average profile width of the common member matrix were identified using the R package “NMF”. Then, the Kaplan–Meier method was utilized to perform overall survival (OS) in different molecular subtypes of the patients. Furthermore, the ESTIMATE and CIBERSORT algorithms were applied to identify the immune microenvironment differences in different molecular subtypes.

### WGCNA and key DE-IRlncRNAs identification

WGCNA is a system biology method to analyze gene expression patterns of multiple samples. It can divide genes with similar expression patterns into the same modules and identify the association between specific traits and modules. To find the module associated with clinical characteristics and clusters, WGCNA was performed using the R package “WGCNA” based on the expression of DE-IRlncRNAs. Then, we selected DE-IRlncRNAs in the most significant module (the grey module) for survival analysis, and ZFPM2-AS1 was determined as the key lncRNA for pan-cancer analysis.

### Differential expression analysis and survival analysis of ZFPM2-AS1 in pan-cancers

To further explore the role of ZFPM2-AS1 in pan-cancers, we used the UCSC Xena database to download the transcription expression data, clinical information, stemness score, and immune subtype data of 33 TCGA GDC tumors. We obtained the gene symbol of transcription expression data from the Ensembl and extracted ZFPM2-AS1 expression levels of 33 cancer types. Then, the comparison of ZFPM2-AS1 between tumor and normal tissues was performed in 18 cancer types (BLCA, BRCA, COAD, CHOL, GBM, ESCA, KIRP, KICH, HNSC, KIRC, LIHC, LUSC, GAC, LUAD, PRAD, THCA, READ, and UCEC), which had more than five associated adjacent noncancerous tissues. Furthermore, based on the median expression level of ZFPM2-AS1, 33 cancer samples were classified into low-expression and high-expression groups. To identify the role of ZFPM2-AS1 in the cancer patients’ survival, the Kaplan–Meier method was utilized to perform OS, progression-free interval (PFI), and disease-specific survival (DSS) analyses in 33 cancer types. *P* < 0.05 was regarded as the threshold.

### Tumor immune microenvironment (TIME), TMB, and MSI analysis

The ESTIMATE algorithm was applied to estimate the stromal score and immune score of tumor samples. We examined the correlation between the expression of ZFPM2-AS1 and the stromal/immune score using the Spearman rank correlation coefficient (*P* < 0.05). Subsequently, the CIBERSORT algorithm was utilized to evaluate the composition ratio of infiltrating immune cells in the TME. We performed correlation analysis to investigate the relationship between ZFPM2-AS1 expression and the proportion of immune cells (*P* < 0.05).

TMB, which refers to the number of somatic mutations contained in a tumor cell, is a promising biomarker for immunotherapy response in patients with tumors. MSI, a predictor to guide immunotherapy, represents a hypermutator phenotype in tumors caused by impaired DNA mismatch repair. We further assessed the relationship between ZFPM2-AS1 expression and TMB, and MSI to investigate whether ZFPM2-AS1 could be a promising target for tumor immunotherapy (*P* < 0.05).

### Drug sensitivity analysis and GSEA analysis

We downloaded gene expression and drug sensitivity data of cancer samples from the CellMiner database. We filtered drug sensitivity data with FDA standard certification and clinical laboratory verification. Then, we identified the correlation between drug sensitivity and ZFPM2-AS1 expression. Besides, to explore the role of ZFPM2-AS1 in pan-cancers, GSEA was carried out for the KEGG pathway using the R package “clusterprofiler”. *P* < 0.05 was regarded as the cutoff.

### The construction of ZFPM2-AS1 co-expression network in GAC

To explore potential mechanisms of ZFPM2-AS1 in GAC, target mRNAs of ZFPM2-AS1 were identified based on correlation coefficient |R| > 0.3 and *P* < 0.001. We further visualized the ZFPM2-AS1-mRNA co-expression network using the Cytoscape software. In addition, we performed the differential analysis and survival analysis on target mRNAs of ZFPM2-AS1 in GAC. NUP107 and C8orf76 were selected for differential expression analysis, survival analysis, TMB, and MSI analysis in pan-cancers.

### TME and immune subtype analysis of NUP107 and C8orf76 in GAC

We evaluated the association between NUP107 and C8orf76 expression and the stromal/immune score of the TME (*P* < 0.05). Then, we intersected transcription expression data with stemness score (DNA methylation-based) (DNAss) and stemness score (RNA expression-based) (RNAss). The relationship between NUP107 and C8orf76 expression and DNAss and RNAss of GAC was identified using the Spearman correlation test. Furthermore, six immune subtypes (C1, wound healing; C2, INF-r dominant; C3, inflammatory; C4, lymphocyte depleted; C5, immunological quiet; and C6, TGF-β dominant) were obtained from the UCSC Xena database. We performed the immune subtype analysis of NUP107 and C8orf76 in GAC using R language loaded with packages “limma,” “ggplot2,” and “reshape2”.

### Cell culture, transfection and real-time polymerase chain reaction (qRT-PCR)

The normal gastric cell line (GES-1) and GC cell lines (AGS, MKN-28, HGC-27, and MKN-45) were obtained from ATCC (Manassas, VA, USA). GES-1 cells were cultured in DMEM medium (Gibco). MKN-28, HGC-27, and MKN-45 were grown in RPMI-1640 medium (Gibco). AGS were cultured in F-12 K medium (Gibco). All mediums were supplemented with 10% fetal bovine serum (Gibco), and 10 mg/mL streptomycin, and 100 U/mL penicillin. The plasmids of lncRNA ZFPM2-AS1 and negative control were purchased from GeneCreate (Wuhan, China). Then, they were transfected into AGS via lipofectamine 2000 (Invitrogen, USA). After 48 h, the transfected cell line was applied for further experiments, and the transfection efficiency was evaluated by qRT-PCR.

Total RNAs in cell lines were extracted using TRIzol (Invitrogen) based on the manufacturer’s protocol. Then, PrimeScript RT Reagent Kit was utilized to synthesize cDNA (Thermo Scientific). qRT-PCR was carried out using SYBR Green Master Mix (Thermo Scientific). The 2^−ΔΔCt^ method was utilized to calculate mRNA expression levels, and GAPDH was used as an internal control. The primer sequences were as follows: ZFPM2-AS1 forward primer: CAATGGGACTAAGCCAGGCA; reverse primer: GGGCTCCACCAACAACCATA; C8orf76 forward primer: 5′-TTATACGAACCAGGCTTCTGC-3′; reverse primer: 5′-GCCAACACACTTCACCTCTG-3′; NUP107 forward primer: 5′-GTCTGTGACACCTGGGAAGA-3′; reverse primer: 5′-CTTGATTCTCTTCCAGAACTCTCT-3′; GAPDH forward primer: 5′-CTCGCTTCGGCAGCACA-3′; reverse primer: 5′-AACGCTTCACGAATTTGCGT-3′.

### Cell counting kit-8 (CCK-8) assay

The proliferative capacity of GC cells was measured using CCK-8 assay. Transfected AGS cells were seeded into 96-well plates at a density of 3 × 10^4^ cells/mL. After incubation for 24 h, 48 h, 72 h, and 96 h, 10 μL CCK-8 (Vazyme, Nanjing, China) was added to each well and absorbance was measured at 450 nm. CCK8 assay also performed to detect the cisplatin sensitivity of GC cells. Transfected AGS cells were inoculated onto 96-well plates overnight and then different concentrations of cisplatin (1, 2, 3, 4, 5, 6, 7, 8 μM) were added to each well for continued culture. After 48 h, cells were incubated with 10 μL CCK-8 for 1 h before the detection of absorbance.

### Statistical analyses

Statistical analyses were performed using Perl (version 5.30.1), GraphPad Prism (version 7.0), and R (version 4.0.2). *P* < 0.05 was defined as statistically significant.

## Results

### DE-IRlncRNAs-based molecular subtypes and TIME analysis

**Fig. 2:**
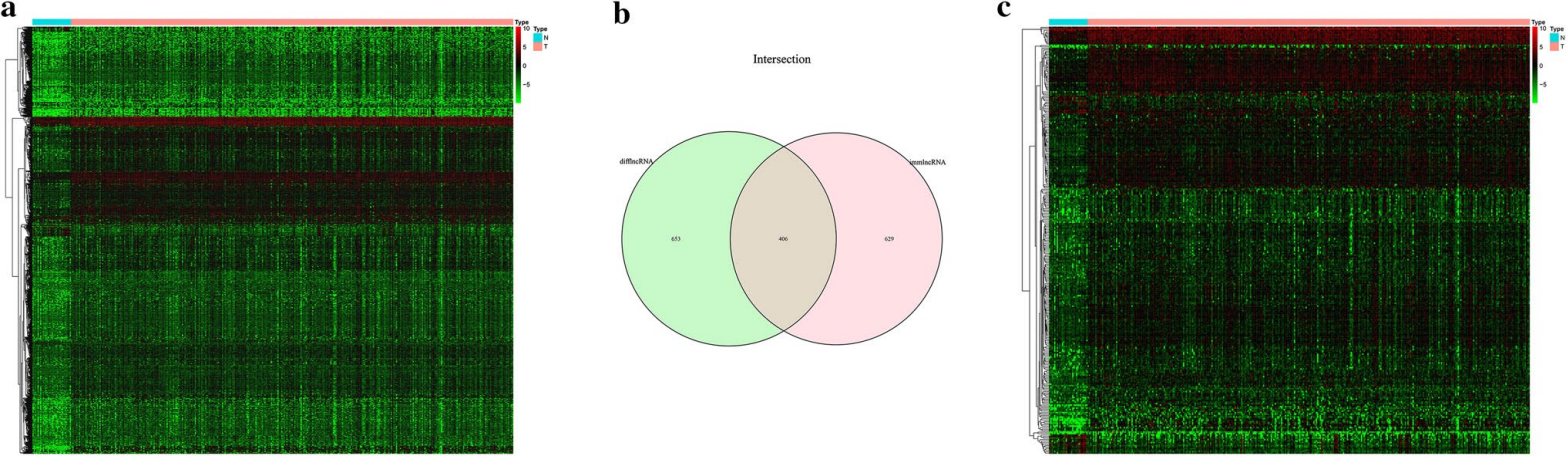
Differentially expressed immune-related lncRNAs. **a** Heatmap of differentially expressed lncRNAs in GAC. **b** Venn diagram of overlapping lncRNAs between immune-related lncRNAs and differentially expressed lncRNAs. **c** Heatmap of differentially expressed immune-related lncRNAs in GAC

**Fig. 3:**
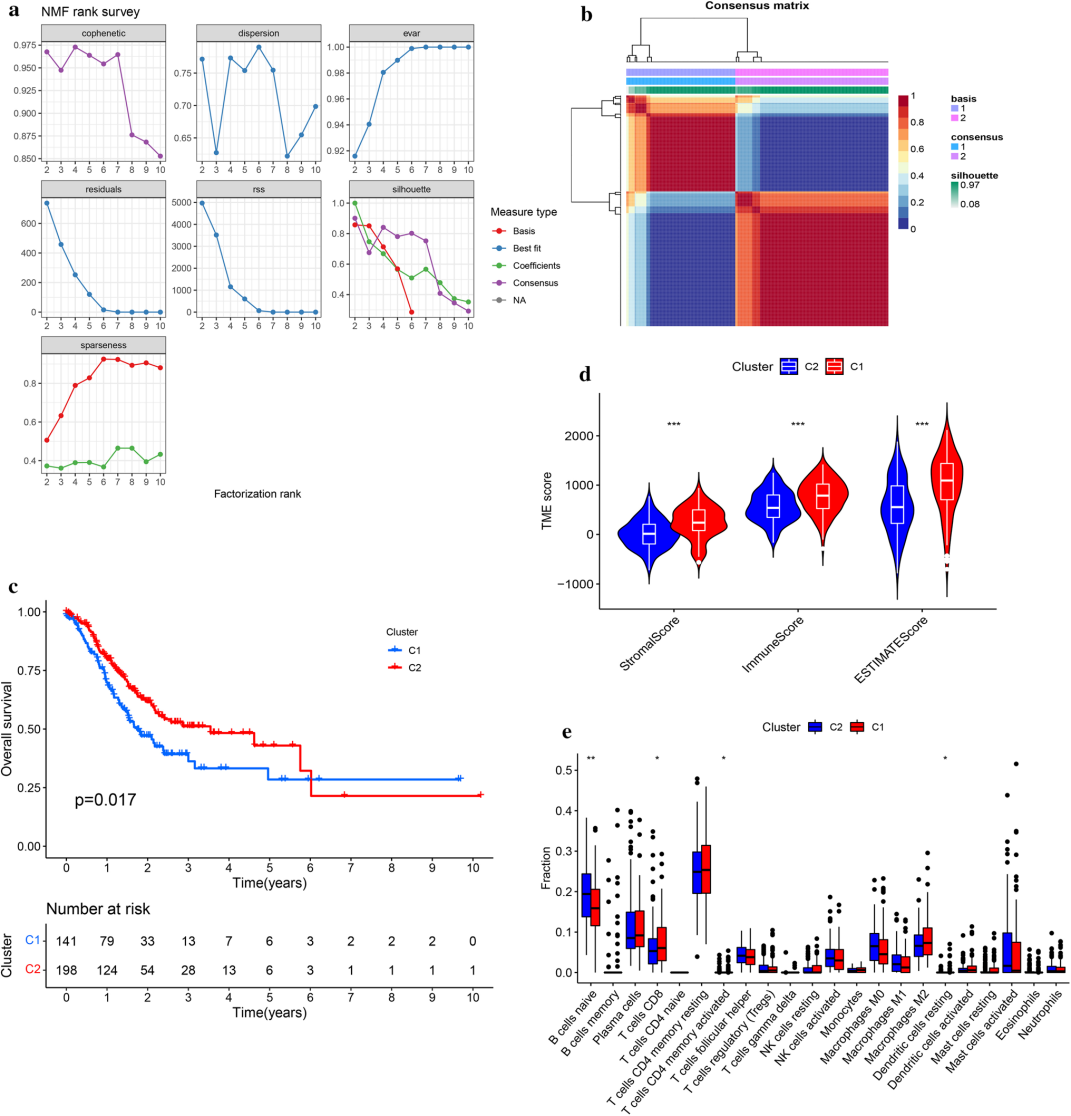
Identification of DE-IRlncRNAs-based molecular subtypes. **a** The cophenetic, rss, and silhouette distributions when the number of clusters was set as 2–10. **b** Consensus map of NMF clustering. **c** The survival curves of 2 molecular subtypes. **d** Comparisons of immune, estimate, and stroma scores between 2 molecular subtypes. **e** Comparisons of infiltrating immune cells between 2 molecular subtypes

The list of 2498 IRGs was obtained from the ImmPort database, and 1035 IRlncRNAs were identified according to the Pearson correlation analysis (|R| > 0.3 and *P* < 0.001). Among them, 406 IRlncRNAs were differentially expressed between the GAC and normal tissues (|Log2FC| > 1 and FDR < 0.05) (Fig. 2). Then, these 406 DE-IRlncRNAs were used for NMF cluster analysis, and the optimal number of clusters was determined as 2 via the comprehensive correlation coefficient (Fig. 3a and b). All GAC patients were divided into two clusters, including Cluster 1 (n = 141) and Cluster 2 (n = 198). The Kaplan–Meier curve showed that Cluster 1 patients had a worse OS rate than Cluster 2 (*P* = 0.017) (Fig. 3c). We further investigated the differences in the immune microenvironment between the two clusters. As shown in Fig. 3d, the immune, estimate, and stromal scores were significantly elevated in Cluster 1 compared with Cluster 2. Furthermore, CIBERSORT revealed that the abundance of T cells CD8, T cells CD4 memory activated, Dendritic cells resting, and B cells naive were significantly different in Cluster 1 and Cluster 2 (*P* < 0.05) (Fig. 3e).

### WGCNA analysis and identification of key DE-IRlncRNAs

To determine the crucial modules related to GAC, WGCNA was carried out based on the expression data of 406 DE-IRlncRNAs. The results of WGCNA revealed that the grey module had the highest correlation with Cluster (r = − 0.3, *P* = 2e−08) and grade (r = 0.25, *P* = 3e−06) (Fig. S1). Then, the Kaplan–Meier method was utilized to evaluate the prognostic value of 125 DE-IRlncRNAs in the grey module. The results showed that 12 DE-IRlncRNAs (AP001107.5, AL365181.3, TNFRSF10A-AS1, AP000695.2, A2M-AS1, AL023803.1, AP000695.1, AC136475.3, AL590666.2, AP003071.4, AC005586.1, and ZFPM2-AS1) in the grey module were associated with the survival of GAC patients (Table 1 and Fig. S2). Finally, ZFPM2-AS1 with the most significant prognostic value was selected for pan-cancer analysis.

**Table 1:** The survival of DE-IRlncRNAs in the grey module

LncRNA	*P*-value
AC012363.1	0.996898
AC006329.1	0.992848
AL356740.3	0.982899
AC005006.1	0.97064
AC008514.1	0.93599
AC024896.1	0.93492
TM4SF1-AS1	0.930508
AC007991.4	0.929411
AL606834.1	0.924328
LHFPL3-AS2	0.903146
KRT7-AS	0.880246
MGC32805	0.876812
AC025857.2	0.820212
CASC19	0.805535
AC005550.2	0.790935
AC104534.1	0.783331
LINC02582	0.777969
AC108451.2	0.776981
AC114488.1	0.766401
AC245884.9	0.751445
FAM201A	0.734365
AC004847.1	0.707329
AC090152.1	0.692489
ST8SIA6-AS1	0.674445
AC000061.1	0.660719
AP003390.1	0.629214
AC009065.2	0.617792
SLCO4A1-AS1	0.610855
AC022150.4	0.610363
TSPEAR-AS2	0.595134
AC068580.1	0.588947
NKILA	0.57245
AC124067.2	0.566155
AL158166.1	0.556415
ABALON	0.551759
LINC00853	0.551556
ZBTB46-AS1	0.534618
AC097639.1	0.523542
KRTAP5-AS1	0.507264
TSPEAR-AS1	0.503464
CASC8	0.494948
AC025580.1	0.489566
UNC5B-AS1	0.485818
MBNL1-AS1	0.476984
AL158206.1	0.470208
AC007566.1	0.463015
AC007991.2	0.459502
ZNF710-AS1	0.456275
PLAC4	0.448398
TGFB2-AS1	0.445334
LINC00659	0.444493
AC125257.1	0.421888
AC104699.1	0.419452
AC092535.4	0.41501
UBXN10-AS1	0.414599
AL450992.2	0.405956
HOXB-AS4	0.39411
AC026369.2	0.389877
AL157871.2	0.384637
AC007996.1	0.380787
FEZF1-AS1	0.379495
U62317.2	0.379144
VPS9D1-AS1	0.371015
AC083809.1	0.363786
C8orf31	0.354306
AL031985.3	0.354071
AC022509.3	0.349437
AGAP2-AS1	0.332588
AC009005.1	0.33228
AC008760.2	0.322189
AC107959.3	0.319053
AC104958.2	0.315825
AC046143.1	0.309472
AL162231.2	0.307844
TMEM132D-AS1	0.298586
AC009065.5	0.292543
TFAP2A-AS1	0.270436
LMO7-AS1	0.266092
AP002954.1	0.259131
AL354993.2	0.252088
POLH-AS1	0.241903
AC005288.1	0.238904
AC100861.1	0.224101
MAFG-DT	0.22249
AL139393.2	0.222253
AC003965.2	0.21472
AF001548.1	0.212054
AC124312.5	0.203998
TYMSOS	0.185426
AL109615.3	0.183556
HOTTIP	0.181509
LINC00982	0.18084
AC053503.4	0.175742
AP003071.3	0.170767
GAS6-AS1	0.165138
AL355388.1	0.155924
AL162595.1	0.15059
AC004264.1	0.145704
AC100803.2	0.142581
AC026356.1	0.128803
HOXC-AS1	0.118058
HOXA11-AS	0.106916
SERTAD4-AS1	0.10349
PCAT7	0.096959
HAND2-AS1	0.090067
MIR100HG	0.089976
PGM5-AS1	0.089709
NALT1	0.08185
RHPN1-AS1	0.081286
AC036108.3	0.071949
BX255925.1	0.065393
HOXA10-AS	0.062563
AC026471.3	0.06221
AP001107.5	0.04948
AL365181.3	0.039352
AP003071.4	0.035313
AP000695.2	0.029232
AL023803.1	0.028807
TNFRSF10A-AS1	0.017539
A2M.AS1	0.01351
AP000695.1	0.011527
AC136475.3	0.01043
AL590666.2	0.009739
AC005586.1	0.001782
ZFPM2-AS1	0.0007

### ZFPM2-AS1 expression analysis and survival analysis in pan-cancers

**Fig. 4:**
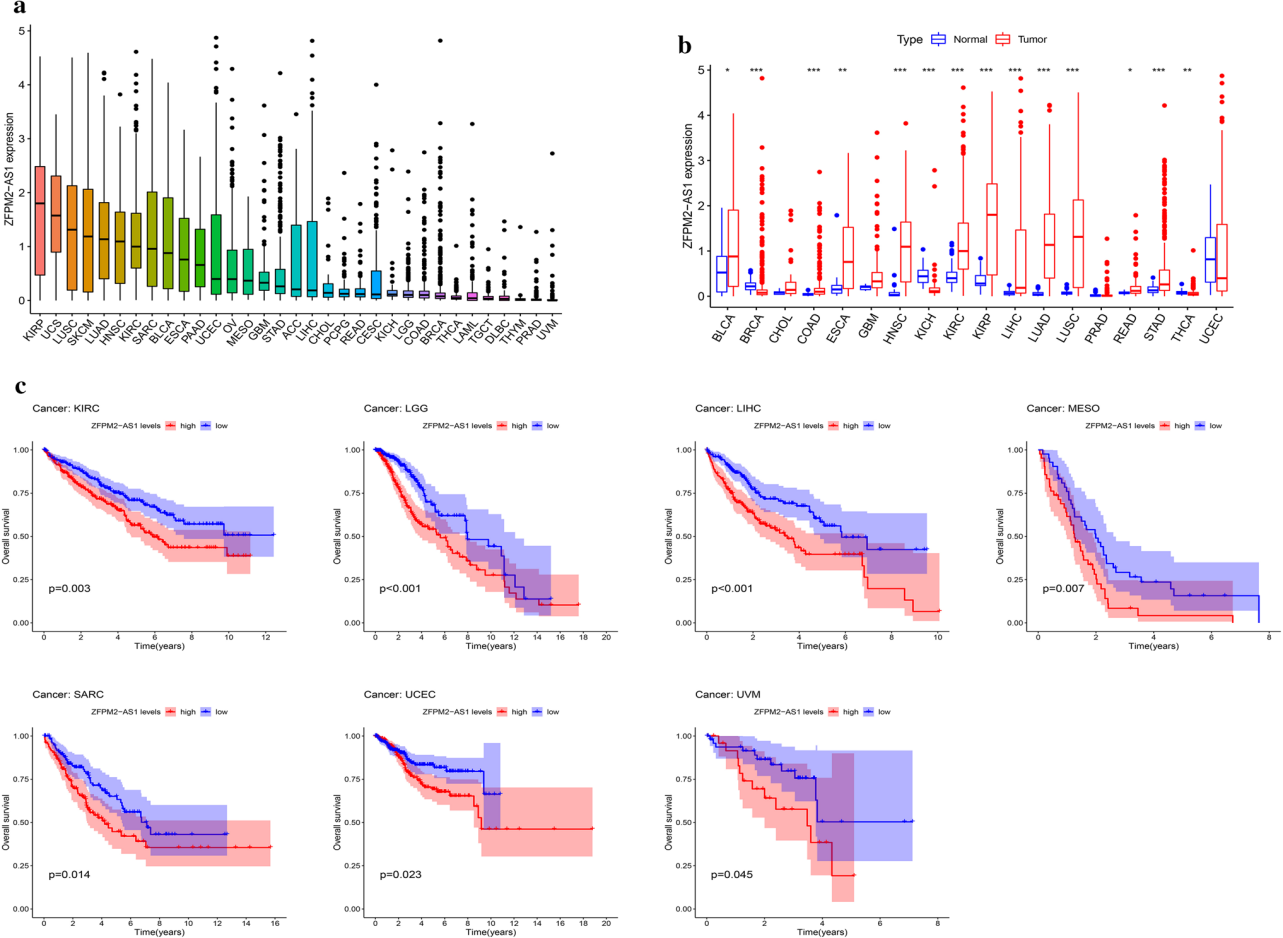
Expression analysis and survival analysis of ZFPM2-AS1 in pan-cancers. **a** Expression level of ZFPM2-AS1 in pan-cancers. **b** Differential expression of ZFPM2-AS1 in pan-cancers. **c** The survival curve of ZFPM2-AS1 for OS in pan-cancers. *P < 0.05, **P < 0.01, ***P < 0.001

**Fig. 5:**
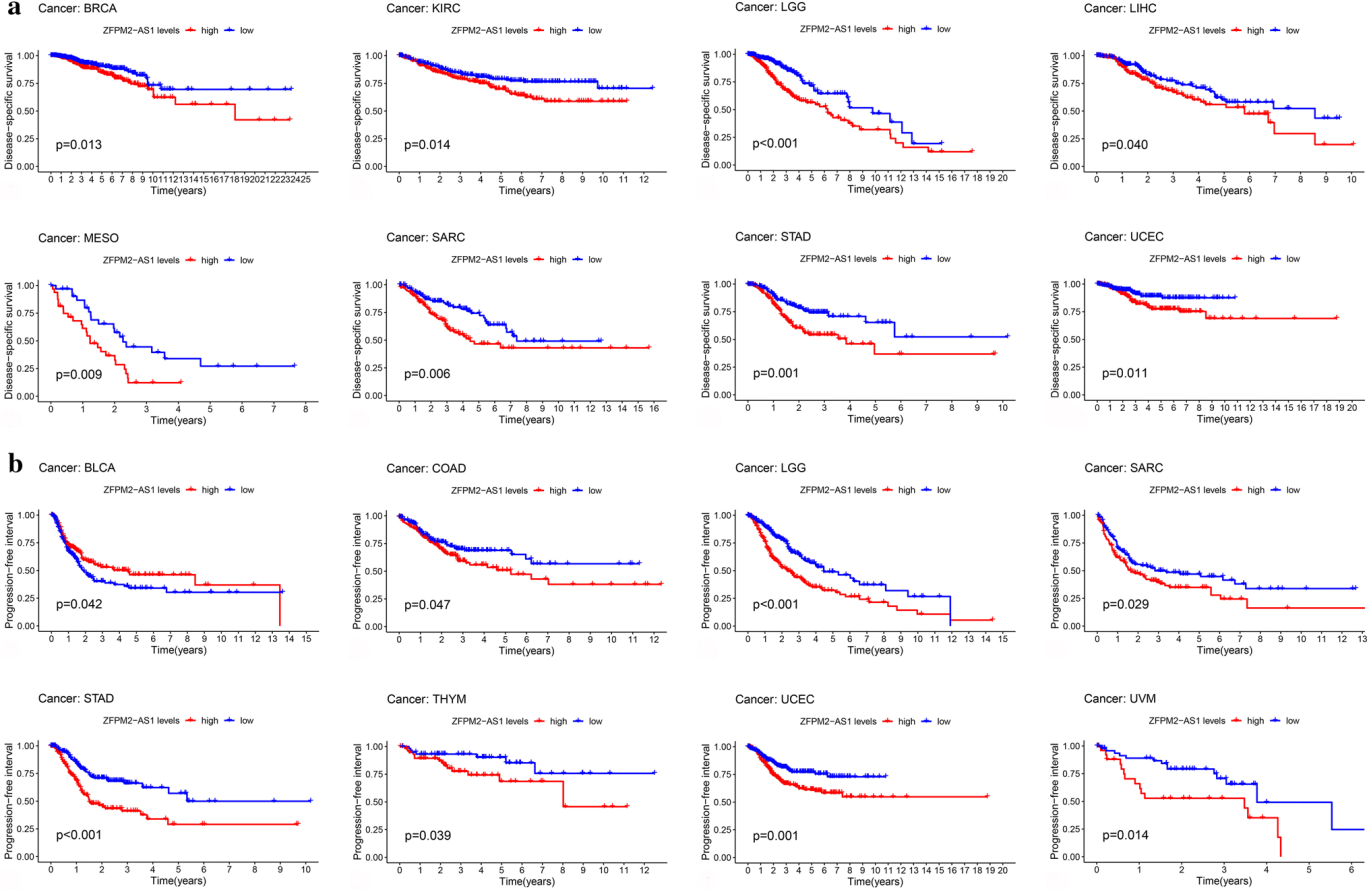
The survival curve of ZFPM2-AS1 for DSS and PFI in pan-cancers. **a** The survival curve of ZFPM2-AS1 for DSS in pan-cancers. **b** The survival curve of ZFPM2-AS1 for PFI in pan-cancers

We assessed the lncRNA levels of ZFPM2-AS1 in 33 cancer types from TCGA. As shown in Fig. 4a and b, ZFPM2-AS1 levels were significantly increased in BLCA, BRCA, COAD, ESCA, KICH, HNSC, KIRP, KIRC, LIHC, LUSC, GAC, LUAD, THCA, and READ. Furthermore, to evaluate the impact of ZFPM2-AS1 in cancer patients’ survival, we divided all samples into high-expression and low-expression groups and compared the OS, DSS, and PFI rates of the 33 cancer types. The Kaplan–Meier curves revealed that ZFPM2-AS1 high expression had a worse OS rate than those with ZFPM2-AS1 low expression in KIRC, LGG, LIHC, MESO, SARC, GAC, UCEC, and UVM (*P* < 0.05) (Fig. 4c). Higher expression levels of ZFPM2-AS1 also indicated worse DSS in BRCA, KIRC, LGG, LIHC, MESO, SARC, GAC, and UCEC, and worse PFI in BLCA, COAD, LGG, SARC, GAC, THYM, UCEC, UVM (*P* < 0.05) (Fig. 5a and b).

### The correlation of ZFPM2-AS1 expression with TIME

**Fig. 6:**
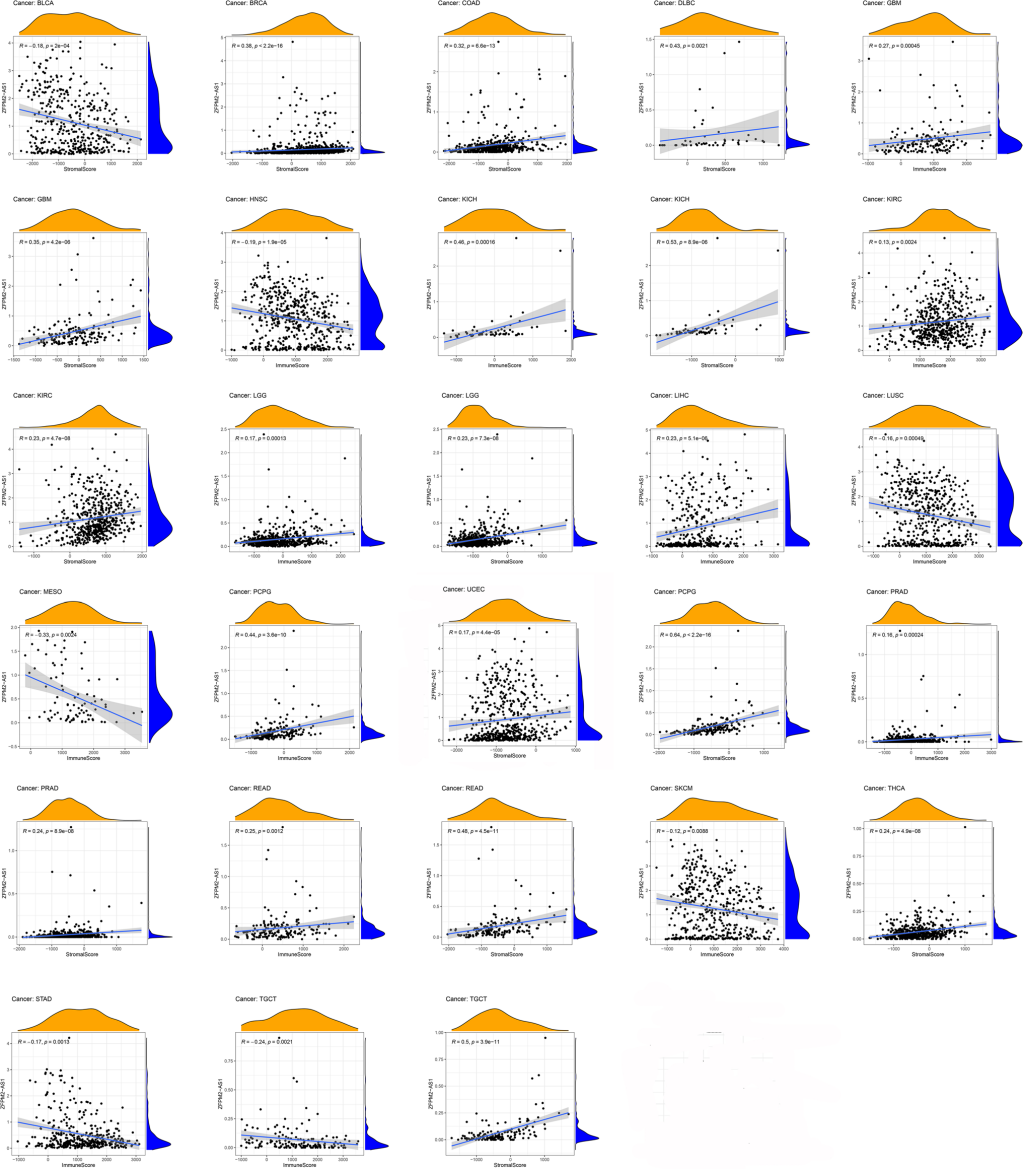
The relationship between ZFPM2-AS1 expression and the stromal/immune score of TME in pan-cancers

We obtained the immune and stromal scores of all tumor samples and assessed the correlation between ZFPM2-AS1 expression and both scores of the TME. We visualized the results at *P* < 0.001. As shown in Fig. 6, the high expression of ZFPM2-AS1 was negatively correlated with the stromal score in BLCA but positively associated with COAD, BRCA, DLBC, KIRC, GBM, KICH, READ, LGG, PRAD, TGCT, PCPG, UCEC, and THCA. The expression of ZFPM2-AS was positively related to the immune score in KICH, GBM, LIHC, KIRC, LGG, PRAD, PCPG, and READ, while negatively associated with MESO, HNSC, GAC, LUSC, SKCM, and TGCT.

We further analyzed the relationship between the level of 22 infiltrating immune cells and ZFPM2-AS1 expression. We visualized the results at *P* < 0.001. The result showed that highly expressed ZFPM2-AS1 was negatively related to B cells naïve in BLCA but positively related to Dendritic cells resting. In BRCA, ZFPM2-AS1 expression was negatively associated with Macrophages M1, T cells CD8, and T cells follicular helper, but positively associated with Mast cells resting and T cells CD4 memory resting. In COAD, the expression of ZFPM2-AS1 was negatively associated with T cells CD8 and NK cells activated but positively associated with Neutrophils. In GBM, the level of ZFPM2-AS expression was positively correlated with Mast cells activated but negatively correlated with Mast cells resting. In HNSC, the expression of ZFPM2-AS1 was negatively associated with B cells naïve, Mast cells resting, T cells CD8 and T cells regulatory (Tregs), but positively associated with Dendritic cells activated, Dendritic cells resting, Macrophages M0, Mast cells activated, and Neutrophils. Besides, high level of ZFPM2-AS1 was positively related to Monocytes in KIRP and Neutrophils in LGG but negatively related to NK cells resting in LIHC and T cells CD8 in LUSC. In SKCM, ZFPM2-AS expression was positively correlated with Macrophages M0, Mast cells activated, T cells CD4 memory resting, T cells gamma delta, but negatively correlated with B cells naïve, Plasma cells, T cells CD8, T cells follicular helper, and Tregs. In TGCT, highly expressed ZFPM2-AS1 was positively associated with Macrophages M2 and Tregs but negatively associated with B cells naïve and T cells CD4 memory activated. Furthermore, the expression of ZFPM2-AS1 had a significant positive relationship with T cells follicular helper in GAC and Macrophages M0 in SARC, while had a significant negative relationship with NK cells activated in PCPG (Fig. S3).

### Immune-related factors, TMB, and MSI, drug sensitivity and GSEA analysis of ZFPM2-AS1 in pan-cancers

**Fig. 7:**
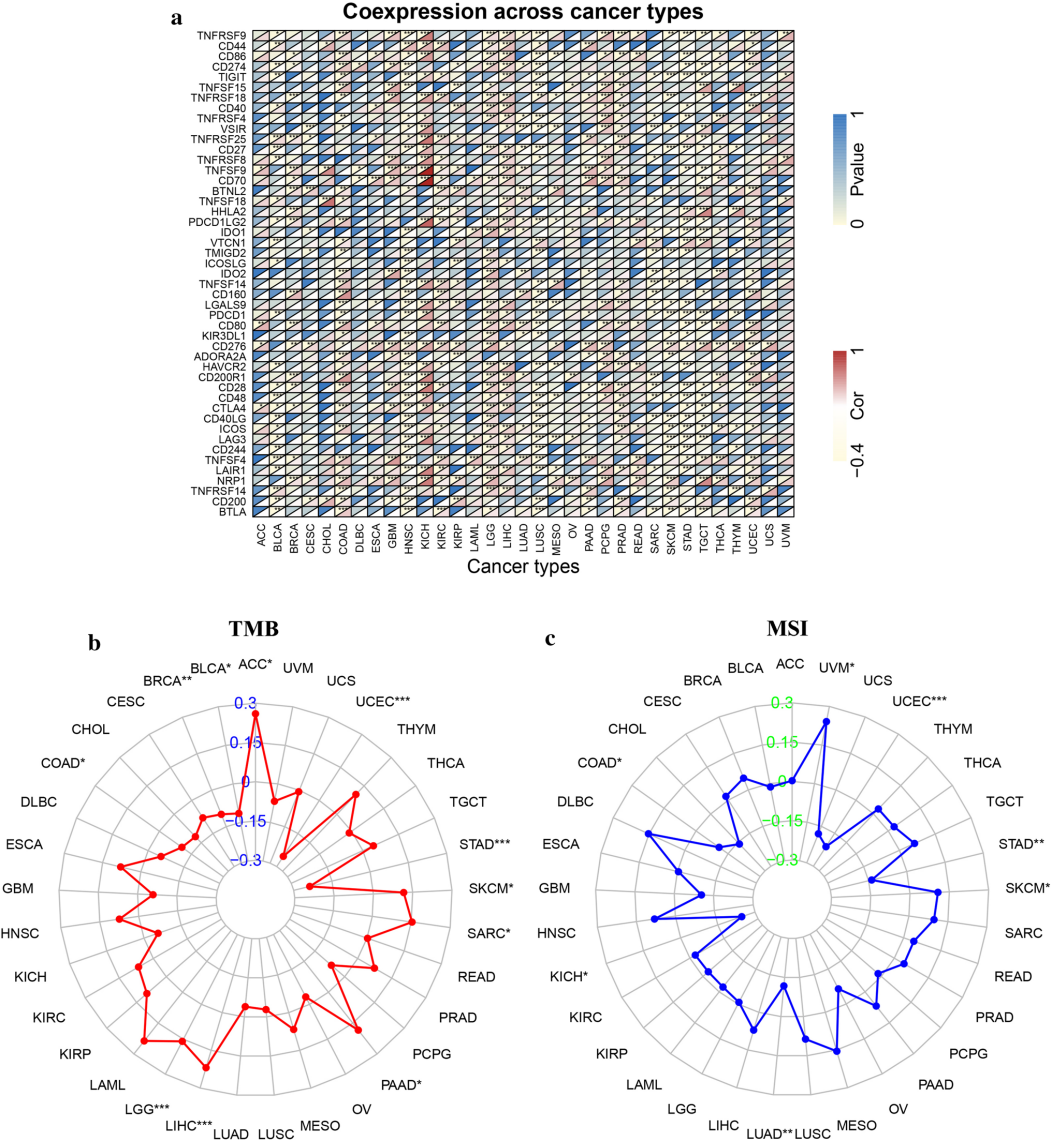
The relationship between ZFPM2-AS1 expression and immune-related factors, TMB, and MSI in pan-cancers. **a** The relationship between ZFPM2-AS1 expression and immune-related factors. **b** The relationship between ZFPM2-AS1 expression and TMB. **c** The relationship between ZFPM2-AS1 expression and MSI. *P < 0.05, **P < 0.01, ***P < 0.001

To explore the immune-related role of ZFPM2-AS in tumors, we analyzed the relationship between ZFPM2-AS1 expression and immune-related factors. Heatmaps showed that ZFPM2-AS1 was correlated with several known immune checkpoints, including CTLA4, PDCD1 (PD-1), CD274 (PD-L1), TIGIT, LAG-3, HAVCR2 (TIM-3), and IDO1, in most tumors (Fig. 7a). We further evaluated the correlation of ZFPM2-AS1 expression with TMB and MSI. As shown in Fig. 7b, ZFPM2-AS1 expression was correlated with TMB in COAD, BRCA, LGG, BLCA, LIHC, SKCM, ACC, PAAD, UCEC, GAC, and SARC. ZFPM2-AS1 expression was correlated with MSI in COAD, UCEC, KICH, UVM, GAC, LUAD, and SKCM (Fig. 7c).

**Fig. 8:**
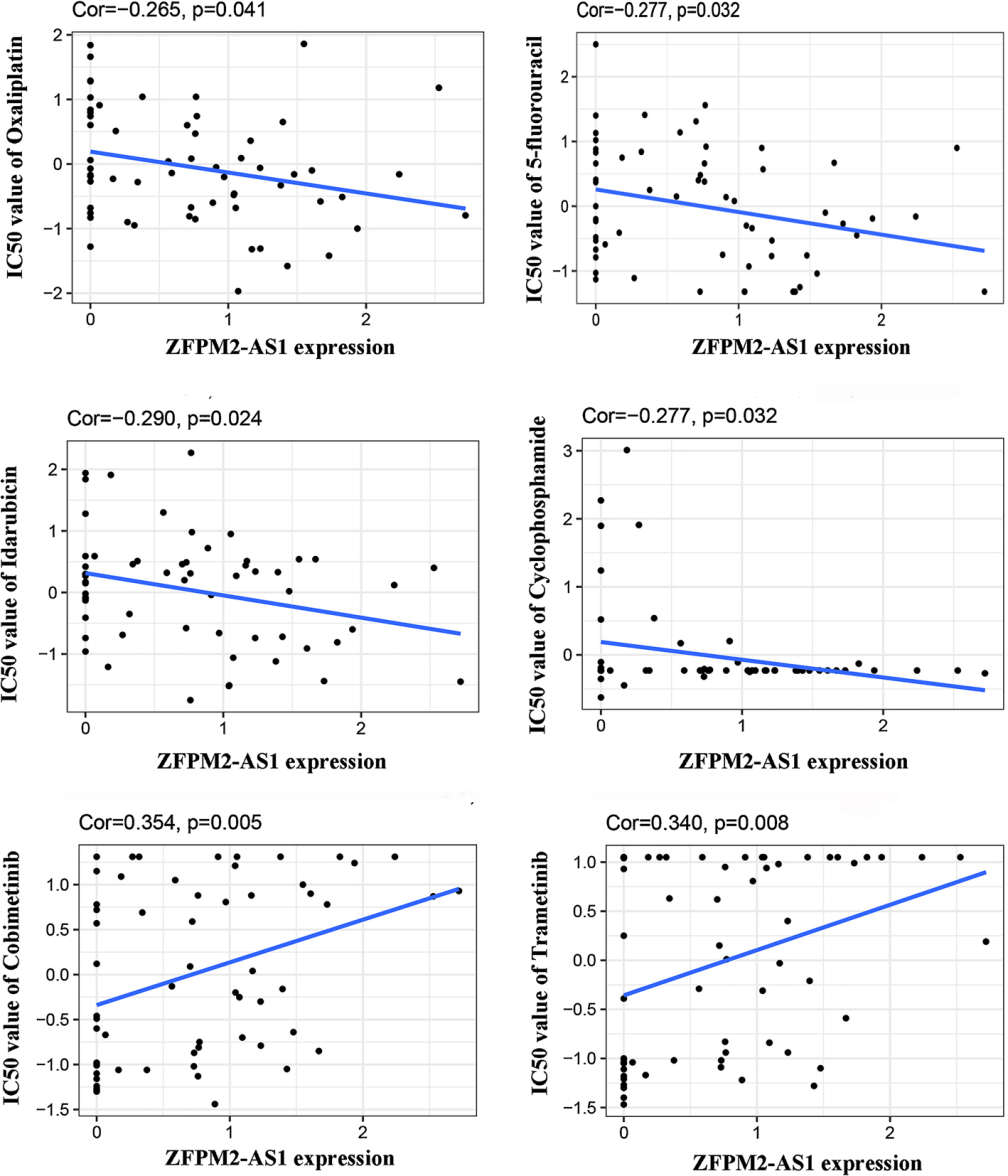
The relationship between ZFPM2-AS1 and anticancer drugs sensitivity in CellMiner

Then, we explored the association between ZFPM2-AS1 and drug sensitivity. The result revealed that ZFPM2-AS1 expression had a positive relationship with the sensitivity of cobimetinib, and trametinib. The expression of ZFPM2-AS1 had a negative relationship with the sensitivity of idarubicin, cyclophosphamide, 5-FU, and oxaliplatin (Fig. 8). Furthermore, to explore the role of ZFPM2-AS1 in various types of cancer, GSEA was performed according to the expression of ZFPM2-AS1. We found that ZFPM2-AS1 in pan-cancer tissues was mainly involved in antigen processing and presentation, cytokine-cytokine receptor interaction, focal adhesion, and cytosolic DNA sensing pathway. Importantly, we found ZFPM2-AS1 in GAC primarily involved in immune-related pathways, including antigen processing and presentation, Toll-like receptor signaling pathway, and RIG-I-like receptor signaling (Fig. S4).

### Construction of ZFPM2-AS1 co-expression network

**Fig. 9:**
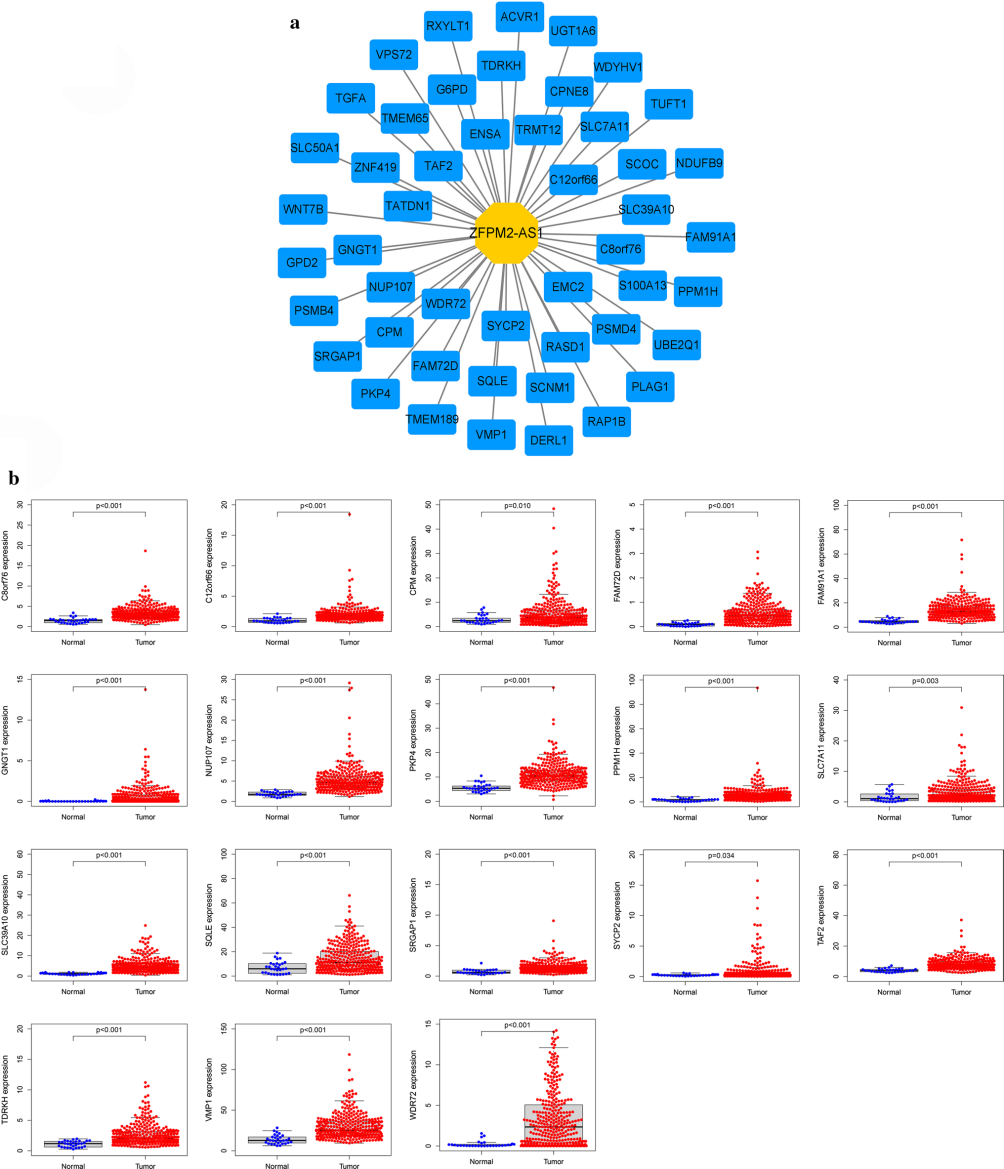
The co-expression network of ZFPM2-AS1 in GAC. **a** Construction of the ZFPM2-AS1-mRNA co-expression network. **b** Differential expression of target mRNAs in GAC samples and normal samples from TCGA

To investigate the underlying mechanisms of ZFPM2-AS1 in GAC, we constructed a ZFPM2-AS1-mRNA co-expression network. As shown in Fig. 9a, a total of 48 genes were positively correlated with ZFPM2-AS1. Among them, NUP107 and C8orf76 were remarkably upregulated in GC samples (Fig. 9b). Similar results were observed in the pairing analysis between GC tissues and adjacent noncancerous tissues from the same patient (Fig. S5). Furthermore, the survival analysis showed that GC patients with NUP107 low expression had a higher survival rate than those with NUP107 high expression. GC patients with C8orf76 low expression also had a higher survival rate than those with C8orf76 high expression (Fig. S6).

### Differential expression analysis, survival analysis, TMB, and MSI analysis of NUP107 and C8orf76 in pan-cancers

**Fig. 10:**
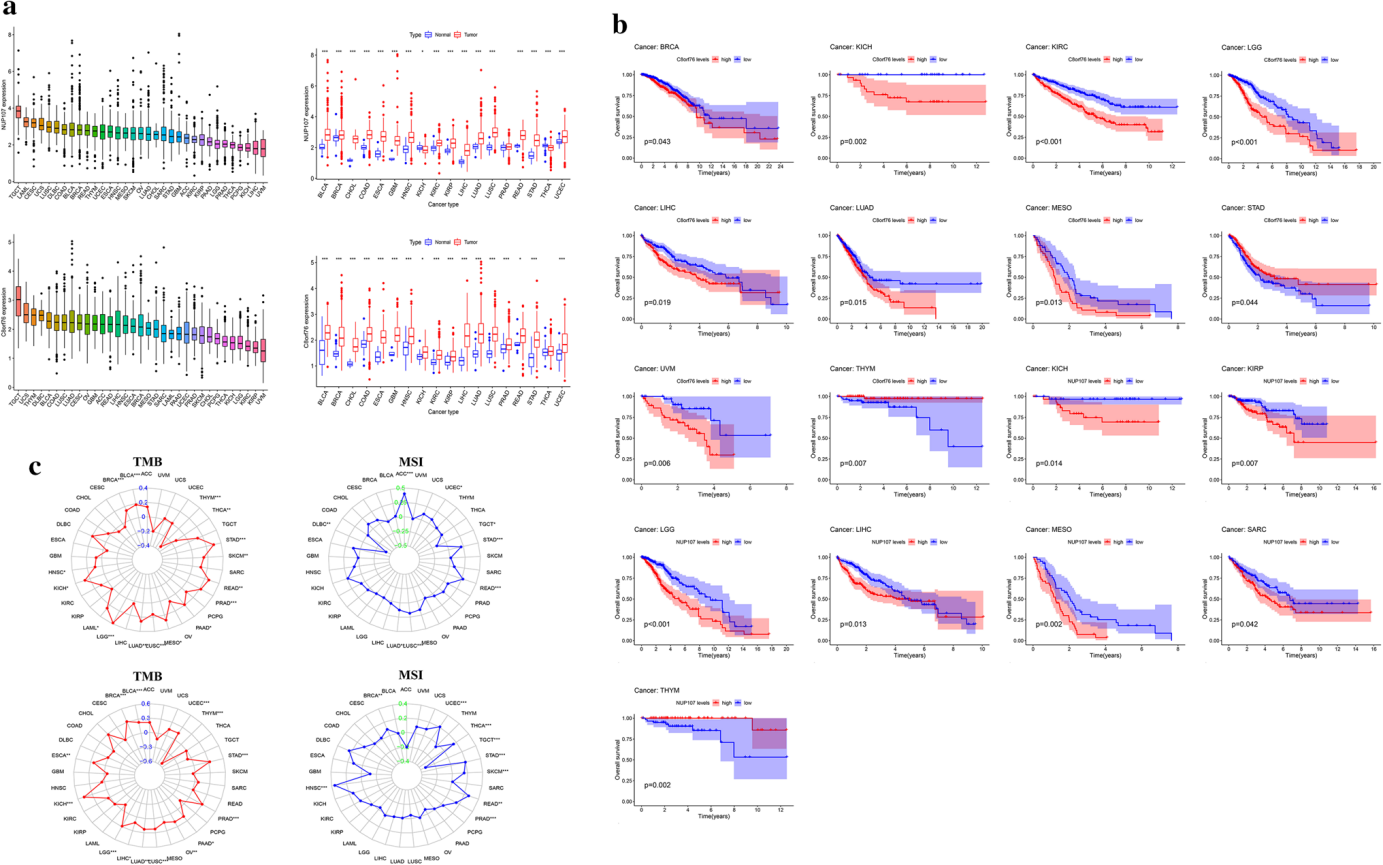
Expression analysis, survival analysis, TMB, and MSI analysis of NUP107 and C8orf76 in pan-cancers. **a** Expression level of NUP107 and C8orf76 in pan-cancers. *P < 0.05, **P < 0.01, ***P < 0.001. **b** The survival curve of NUP107 and C8orf76 for OS in pan-cancers. **c** The correlation between NUP107 and C8orf76 expression and TMB, and MSI in pan-cancers

We further evaluated the difference of NUP107 and C8orf76 expression in the other 32 types of tumors profiled in TCGA. The result revealed that NUP107 was significantly differentially expressed in 15 cancer types. Upregulated expression of NUP107 was observed in 13 types of cancer (BLCA, BRCA, CHOL, COAD, ESCA, GBM, HNSC, KIRC, KIRP, LIHC, LUAD, LUSC, PRAD, READ, and UCEC), and downregulated expression of NUP107 was observed in two types of cancer (KICH and THCA) (Fig. 10a). The expression of C8orf76 was remarkably upregulated in 16 types of cancer (BLCA, BRCA, CHOL, COAD, ESCA, GBM, HNSC, KICH, KIRC, KIRP, LIHC, LUAD, LUSC, PRAD, READ, and UCEC) (Fig. 10a).

Subsequently, to probe the effect of NUP107 and C8orf76 in cancer patients’ survival, we divided all samples into high-expression and low-expression groups and compared the OS rate of the other 32 cancer types. As shown in Fig. 10b, the high expression of NUP107 had a worse OS rate than the low expression of NUP107 in KICH, KIRP, LGG, LIHC, SARC, MESO, and THYM. The high expression of C8orf76 had a worse OS rate than the low expression of C8orf76 in BRCA, KICH, KIRC, LGG, LIHC, LUAD, MESO, THYM, and UVM. Furthermore, TMB and MSI analyses were carried out to investigate whether NUP107 and C8orf76 could be promising immunotherapy targets. As shown in Fig. 10c, NUP107 and C8orf76 expression was related to TMB in GAC, THYM, LUSC, BLCA, LUAD, PRAD, BRCA, KICH, PAAD, and LGG. NUP107 and C8orf76 expression was correlated with TMB in UCEC, READ, GAC, and TGCT.

### The correlation of NUP107 and C8orf76 expression with TME and immune subtype of GAC

**Fig. 11:**
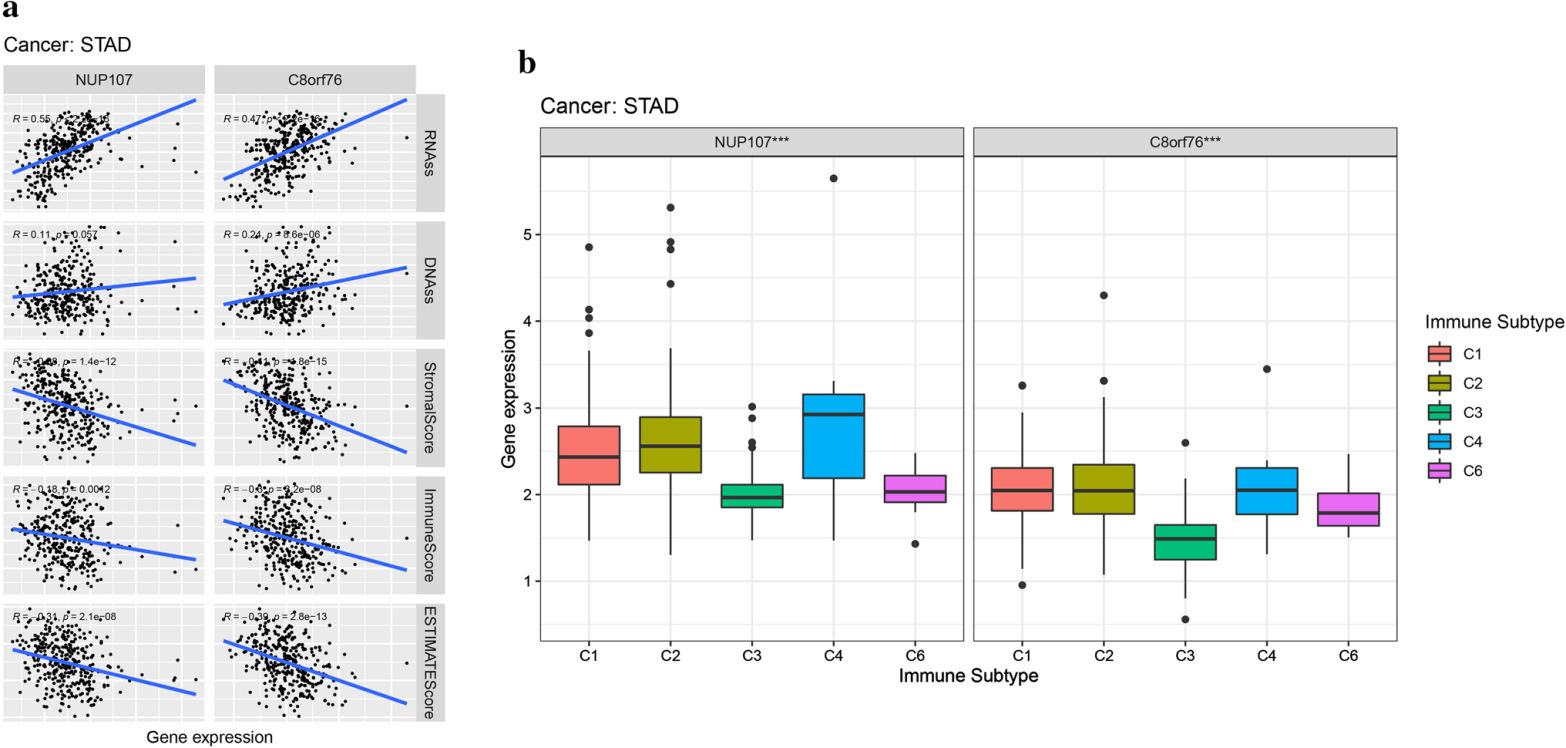
Correlation analysis of NUP107 and C8orf76 expression and GAC microenvironment, stem cell scores, and immune subtype. **a** The correlation between NUP107 and C8orf76 expression and RNAss, DNAss, immune score, estimate score, and stroma score. **b** The correlation between NUP107 and C8orf76 expression and immune subtype. *P < 0.05, **P < 0.01, ***P < 0.001

We performed correlation analysis to determine the association between NUP107 and C8orf76 expression and DNAss, RNAss, stromal score, and immune score of GAC. As shown in Fig. 11a, NUP107 expression was positively associated with RNAss of GAC, while C8orf76 expression was significantly associated with RNAss and DNAss of GAC. Figure 11a also showed that the expression of NUP107 and C8orf76 had a negative relationship with the stromal score and immune score of GAC. We also conducted immune subtype analysis to investigate the relationship between NUP107 and C8orf76 expression and immune infiltrate types in the TME of GAC. The result revealed that the expression of NUP107 and C8orf76 was significantly different in the immune subtype (*P* < 0.001) (Fig. 11b).

### Validation of ZFPM2-AS1, NUP107 and C8orf76 by qRT-PCR

**Fig. 12:**
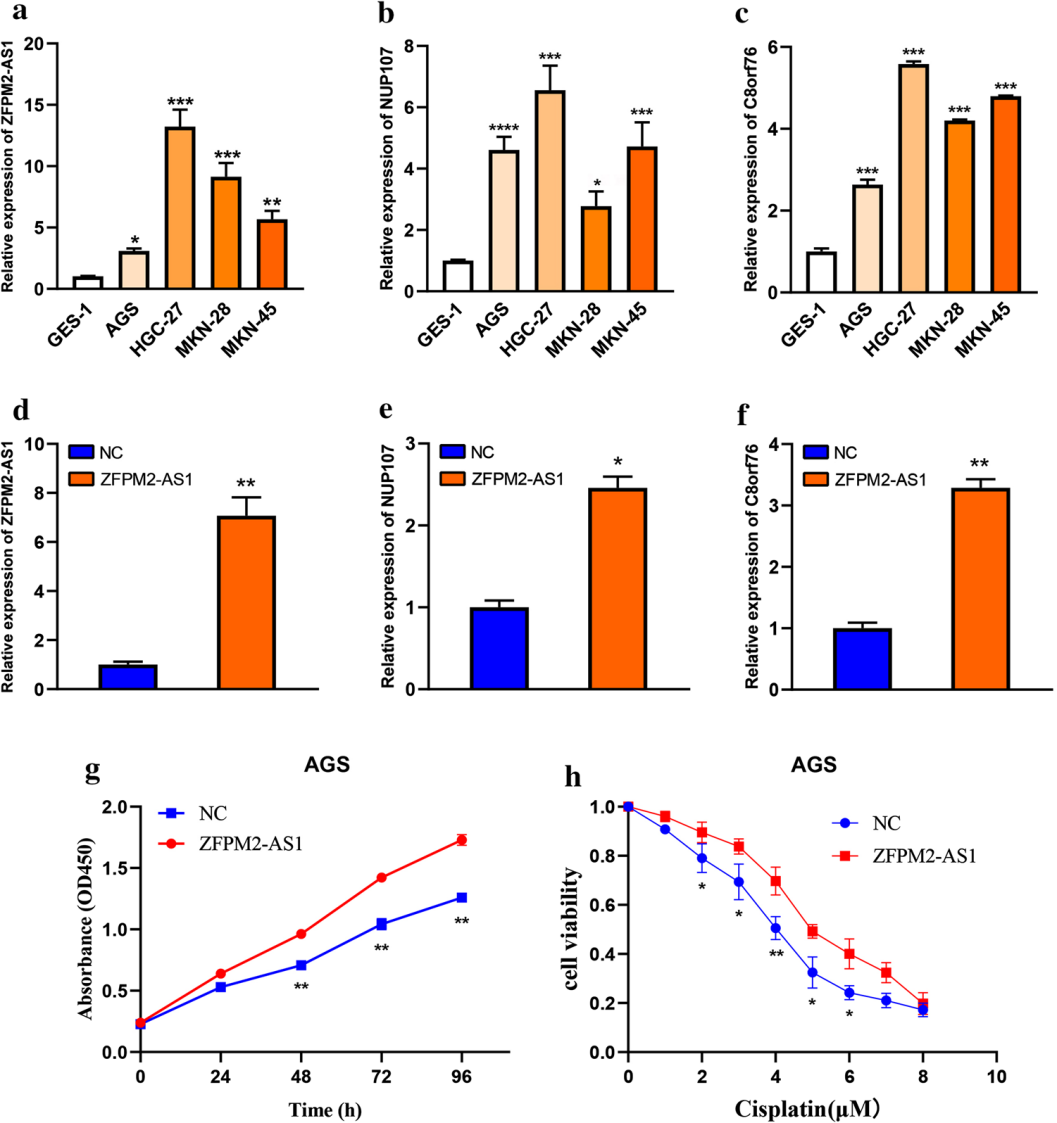
The expression of ZFPM2-AS1, NUP107, and C8orf76 in GC cells. **a** qRT-PCR validation of ZFPM2-AS1 expression in GC cells. **b** qRT-PCR validation of NUP107 expression in GC cells. **c** qRT-PCR validation of C8orf76 expression in GC cells. **d** ZFPM2-AS1 expression in AGS cells after transfecting ZFPM2-AS1 plasmid. **e** NUP107 expression in AGS cells after overexpressing ZFPM2-AS1. **f** C8orf76 expression in AGS cells after overexpressing ZFPM2-AS1. **g** The proliferation capacity of AGS cells after ZFPM2-AS1 overexpression. **h** The sensitivity to cisplatin in AGS cells after ZFPM2-AS1 overexpression. *P < 0.05, **P < 0.01, ***P < 0.001

We performed qRT-PCR in GC cells to validate ZFPM2-AS1, NUP107 and C8orf76 expression levels. The results showed that ZFPM2-AS1, NUP107 and C8orf76 expression were significantly highly expressed in GC cells compared with normal gastric cells (Fig. 12a–c). The expression levels of NUP107 and C8orf76 were consistent with the results of our bioinformatics analysis. Furthermore, we detected NUP107 and C8orf76 expression in AGS cells after transfecting the overexpression plasmid of ZFPM2-AS1. The result of qRT-PCR showed that ZFPM2-AS1 positively regulated NUP107 and C8orf76 expression in AGS cells (Fig. 12d–f). These results suggested that NUP107 and C8orf76 might be potential targets of ZFPM2-AS1. Collectively, these findings further demonstrated that the bioinformatics analysis was reliable.

### The effect of ZFPM2-AS1 on the proliferation and cisplatin sensitivity of GC cells

To explore the role of ZFPM2-AS1 in GC cell proliferation and cisplatin sensitivity, ZFPM2-AS1 overexpression plasmid was transfected into AGS cells. Then, the result of CCK-8 assay revealed that highly expressed ZFPM2-AS1 significantly promoted the proliferation of AGS cells (Fig. 12g). Furthermore, we investigated whether cisplatin sensitivity of AGS cells was regulated by ZFPM2-AS1. The results indicated that overexpression of ZFPM2-AS1 reduced the sensitivity to cisplatin in AGS cells. Our finding suggested that high expression levels of ZFPM2-AS1 may be resistant to cisplatin treatment (Fig. 12h).

## Discussion

LncRNAs are multifunctional molecules that play indispensable roles in the pathogenesis of numerous human genetic diseases, including cancer. In the past few decades, various lncRNAs have been found to be abnormally expressed in different tumor cells. Their role in modulating tumors also has been studied to varying degrees [25, 26]. ZFPM2-AS1, located in 8q23.1, plays an oncogenic role in several tumors. For example, Han et al. detected that ZFPM2-AS1 is highly expressed in lung adenocarcinoma (LUAD) cells. Mechanistically, ZFPM2-AS1 destabilizes ZFPM2 via interacting with UPF1 to facilitate proliferation, and invasion of LUAD cells [10]. Yan et al. also found that ZFPM2-AS1 is significantly increased in small cell lung cancer (SCLC) cells and tissues. Downregulation of ZFPM2‑AS1 attenuates the migration, proliferation, and invasion viability of SCLC cells via the miR-3612/TRAF4 axis [27]. Based on the data from qRT-PCR analysis and TCGA database, ZFPM2-AS1 is recognized as an oncogenic lncRNA in HCC. High levels of ZFPM2-AS1 promote HCC cell cycle progression, cell proliferation, and invasion through miR-653/GOLM1 [11]. Furthemore, a recent study indicated that ZFPM2-AS1 promote thyroid cancer progression via sponging miR-515-5p to modulate TUSC3 expression [12]. However, the underlying molecular mechanisms of dysregulated ZFPM2-AS1 in most tumors remain unclear. In this study, we applied the NMF algorithm to identify a new cluster of GAC, which was correlated with survival and had significant differences in the TIME. Then, ZFPM2-AS1 was identified associated with clusters and prognosis of GAC patients via Weighted Gene Co-Expression Network Analysis (WGCNA) and survival analysis. Furthermore, we first revealed that ZFPM2-AS1 was upregulated in 14 types of cancer, suggesting that ZFPM2-AS1 might be an oncogenic lncRNA in pan-cancers. According to Kaplan–Meier survival curves, ZFPM2-AS1 was also found to be associated with the survival of patients in most tumor types. These results implied that ZFPM2-AS1 might be a promising biomarker for cancer diagnosis and prognosis.

Recently, TME is emerging as a crucial factor in multiple stages of cancer progression, specifically distant metastasis, immune escape, and local resistance, thus affecting the development of frontline interventions in clinical oncology [28]. Importantly, immune cells, as indispensable non-tumor cells in the TME, are considered to be of great value in the diagnosis, prognosis, and treatment of tumors [17, 29, 30]. Our results showed that the level of ZFPM2-AS expression was significantly associated with multiply infiltrating immune cells in the microenvironment of most tumors. Based on these results, we collected 47 common immune-related genes further to explore the immune-related role of ZFPM2-AS in tumors. PDCD1 (PD-1)/CD274 (PD-L1) and CTLA-4 are the hottest immune checkpoints in the current studies. PD-1 is an inhibitory cell surface receptor, which can recognize and bind to PD- L1. The binding of PD-1 to its ligands PD-L1 inhibits the activation of T cells and interrupts the immune surveillance of the TME, leading to tumor immune escape [31, 32]. According to our findings, the expression of ZFPMA-AS1 was highly correlated with PD-1 and PD-L1 in several tumor types. As an inhibitory receptor expressed mainly by T cells, CTLA-4 functions to impair the activation of T cells and is overexpressed upon T cell activation [33]. CTLA-4 is also constitutively expressed on regulatory T cells, which facilitates immune suppression in the TME [34, 35]. Here, we found that the expression of ZFPMA-AS1 was remarkably associated with CTLA-4 in most type of tumors. Furthermore, TIGIT, LAG-3, HAVCR2 (TIM-3), and IDO1 are known as novel immune checkpoint targets that can suppress the activation of effector T lymphocytes, hence mediating a tumor immune escape mechanism [32, 36, 37]. In our study, these novel immune checkpoints were also highly correlated with ZFPMA-AS1expression in most tumors. These findings further implied that ZFPMA-AS1 might affect the progression of cancer by regulating immune-active status in the TME and could be a promising immune target for cancer therapy.

Chemotherapy can improve the survival of patients with locally advanced metastatic or unresectable GC, which plays a vital role in the comprehensive treatment of tumors [22]. However, the development of chemoresistance becomes a major challenge, resulting in the failure of clinical treatment. In recent studies, lncRNAs play an essential role in regulating the drug sensitivity of GC cells. For example, Zhang et al. discovered that CRNDE is associated with the chemosensitivity of GC patients. Mechanistically, CRNDE reduces chemoresistance of GC cells to 5-FU through SRSF6-modulated selective splicing of PICALM [38]. Wu et al. found that FOXD1-AS1 induces cisplatin resistance in GC via activating PIK3CA/PI3K/AKT/mTOR signaling [39]. In addition, Lin et al. revealed that upregulated LINC00200 enhances the chemoresistance of GC cells to oxaliplatin via the E2F1/RAD51 axis [40]. To investigate the indicator function of ZFPMA-AS1 in anticancer drug selection, we analyzed the relationship between anticancer drug sensitivity and ZFPMA-AS1 expression. We found that the expression of ZFPMA-AS was significantly related to the first-line chemotherapeutic drugs, such as 5-FU and oxaliplatin. Our study provided a novel insight for exploring the treatment of tumors and avoiding the resistance of tumors. Furthermore, GSEA further determined that ZFPM2-AS1 in GAC is primarily involved in immune-related pathways, including antigen processing and presentation, Toll-like receptor signaling pathway, and RIG-I-like receptor signaling.

To further explore potential mechanisms of ZFPM2-AS1 in GAC, We constructed a co-expression network to identify target mRNAs. NUP107 and C8orf76 were identified as target mRNAs. NUP107, located on the nuclear rim, is a crucial component of the nuclear pore complex [41, 42]. Recently, NUP107 was reported to participate in the progression of several cancers. For example, Shi et al. demonstrated that the expression of NUP107 is significantly upregulated in cervical cancer samples and cell lines. Besides, overexpression of NUP107 promotes the sensitivity of cervical cancer cells to oxidative insults [43]. Alanee et al. discovered that a single nucleotide variant in NUP107 might be predictive of chemosensitivity in ovarian cancer patients [44]. Based on the bioinformatics analysis, NUP107 is identified as a pivotal gene involved in pancreatic ductal adenocarcinoma (PDAC), which may be a molecular marker for the early diagnosis [45]. Recently, Nong et al. discovered that NUP107, as abridge genes of hepatitis B virus infection-related HCC, is significantly associated with low survival rates of patients [46]. C8orf76, located on chromosome 8q24.13, is a nuclear protein-coding gene [47]. Data from the Human Protein Atlas reveal that upregulated C8orf76 is correlated with the poor prognosis in endometrial cancer, breast cancer, and renal cancer (https://www.proteinatlas.org/ENSG00000189376-C8orf76/pathology). Recent studies reported C8orf76 as a tumor-promoting factor that promotes cell proliferation and metastasis of GC cells. Mechanistic evidence revealed that C8orf76 directly bind to DUSP5P1 promoter region to activate its transcription, therefore inducing the MAPK/ERK signaling pathway [48]. In our study, NUP107 and C8orf76 were found to be overexpressed in GAC and significantly associated with the survival of GAC patients. These findings further implied that NUP107 and C8orf76 might be involved in the development and prognosis of GAC.

To further identify the role of NUP107 and C8orf76 in the TME of GAC, we explore the relationship between NUP107 and C8orf76 expression and DNAss, RNAss, stromal score, and immune score of GAC. The results revealed that NUP107 and C8orf76 expression was significantly associated with immune cells in the GAC microenvironment. Immune type analysis also proved that NUP107 and C8orf76 expression was significantly different in the immune subtype. More importantly, qRT-PCR revealed that NUP107 and C8orf76 expression levels were consistent with the results of bioinformatics analysis. Based on these results, we speculated that ZFPM2-AS1 might play an immunosuppressive role in the microenvironment of GAC by regulating the expression of NUP107 and C8orf76. However, more experiments are needed to confirm our prediction.

## Conclusions

In conclusion, we applied the NMF algorithm to identify a new cluster of GAC, which was correlated with survival and had significant differences in the TIME. Then, ZFPM2-AS1 was identified associated with clusters and prognosis of GAC patients via WGCNA and survival analysis. We further performed a comprehensive analysis of ZFPM2-AS1, revealing its promising role as an indicator of cancer patients’ survival. Meanwhile, ZFPMA-AS1 was found to be involved in regulating the TIME and associated with anticancer drug sensitivity. We speculated that ZFPMA-AS1 might affect the progression of cancer by regulating immune-active status in the TME and could be an immune target for cancer therapy. Furthermore, we constructed a co-expression network and identified potential target mRNAs (NUP107 and C8orf76) of ZFPM2-AS1 in GAC. More interestingly, NUP107 and C8orf76 might play a significant role in the TIME of GAC and could be a potential biomarker for cancer prognosis. Further experiments verified that ZFPM2-AS1, NUP107 and C8orf76 are highly expressed in GC cells and high levels of ZFPM2-AS1 promote the proliferation of GC cells and reduce the sensitivity to cisplatin.

## Supplementary Information

Supplementary file 1 (PDF 21189 KB)

## Data Availability

All data in our study were available from the TCGA database (http://cancergenome.nih.gov/).

## Abbreviations

LncRNAs: Long non-coding RNAs
NMF: Non-negative matrix factorization
TME: Tumor microenvironment
TAMs: Tumor-associated macrophages
GAC: Gastric adenocarcinoma
WGCNA: Weighted gene co-expression network analysis
TMB: Tumor mutational burden
MSI: Microsatellite instability status
DE-IRlncRNAs: Differentially expressed immune-related lncRNAs
IRGs: Immune-related genes
IRlncRNAs: Immune-related lncRNAs
DElncRNAs: Differentially expressed lncRNAs
5-FU: 5-Fluorouracil
CCK-8: Cell counting kit-8
BLCA: Bladder urothelial carcinoma
BRCA: Breast invasive carcinoma
COAD: Colon adenocarcinoma
CHOL: Colon adenocarcinoma
GBM: Glioblastoma multiforme
ESCA: Esophageal carcinoma
KIRP: Kidney renal papillary cell carcinoma
KICH: Kidney chromophobe
HNSC: Head and neck squamous cell carcinoma
KIRC: Kidney renal clear cell carcinoma
LIHC: Liver hepatocellular carcinoma
LUSC: Lung squamous cell carcinoma
LUAD: Lung adenocarcinoma
PRAD: Prostate adenocarcinoma
THCA: Thyroid carcinoma
READ: Rectum adenocarcinoma
UCEC: Uterine corpus endometrial carcinoma
ACC: Adrenocortical carcinoma
CESC: Cervical squamous cell carcinoma and endocervical adenocarcinoma
ESCA: Esophageal carcinoma
DLBC: Lymphoid neoplasm diffuse large B-cell lymphoma
LAML: Acute myeloid leukemia
LGG: Brain lower grade glioma
PCPG: Pheochromocytoma and paraganglioma
MESO: Mesothelioma
PAAD: Pancreatic adenocarcinoma
OV: Ovarian serous cystadenocarcinoma
SARC: Sarcoma
UVM: Uveal melanoma
SKCM: Skin cutaneous melanoma
TGCT: Testicular germ cell tumors
THYM: Thymoma
UCS: Uterine carcinosarcoma
OS: Overall survival
PFI: Progression-free interval
DSS: Disease-specific survival
TIME: Tumor immune microenvironment
