# Supplementary material for: Comprehensive analysis of ZFPM2-AS1 prognostic value, immune microenvironment, drug sensitivity, and co-expression network: from gastric adenocarcinoma to pan-cancers
Source: Discov Oncol. 2022 Apr 13;13:24. doi: 10.1007/s12672-022-00487-0 (PMC9008104; doi:10.1007/s12672-022-00487-0)

Comprehensive Analysis of ZFPM2-AS1 Prognostic Value, Immune Microenvironment, Drug Sensitivity, and Co-expression Network: From Gastric Adenocarcinoma to Pan-cancers

Discover Oncology

Di Chen[1], Mengmeng Wang[1], Xin Jiang[1], Zhifan Xiong[1]

[1] Department of Gastroenterology, Liyuan Hospital, Tongji Medical College, Huazhong University of Science and Technology, Wuhan, China.

Corresponding author: Zhifan Xiong

Department of Gastroenterology, Liyuan Hospital, Tongji Medical College, Huazhong University of Science and Technology, Wuhan, 430061, China.

Email: xiongzhifan@126.com

Fig. S1 Weighted Gene Co-Expression Network Analysis of GAC. (a) Analysis for various soft-thresholding powers. (b) Cluster diagram of gene cluster of GAC. (c) Heatmap of the correlation between the clinical traits and modules.

Fig. S2 The survival curve of differentially expressed immune-related lncRNAs for OS in GAC.

Fig. S3 The relationship between ZFPM2-AS1 expression and infiltrating immune cells in the microenvironment of pan-cancers.

Fig. S4 Gene set enrichment analysis of ZFPM2-AS1 in pan-cancers.

Fig. S5 Differential expression of target mRNAs in paired GAC samples and normal samples from TCGA.

Fig. S6 The survival curve of target mRNAs for OS in GAC.

Supplementary Figure 1

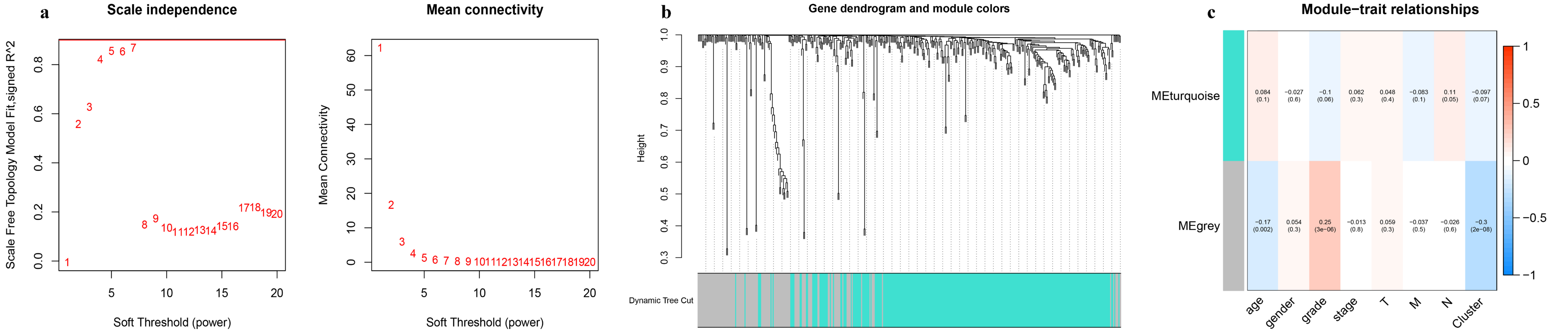

Supplementary Figure 2

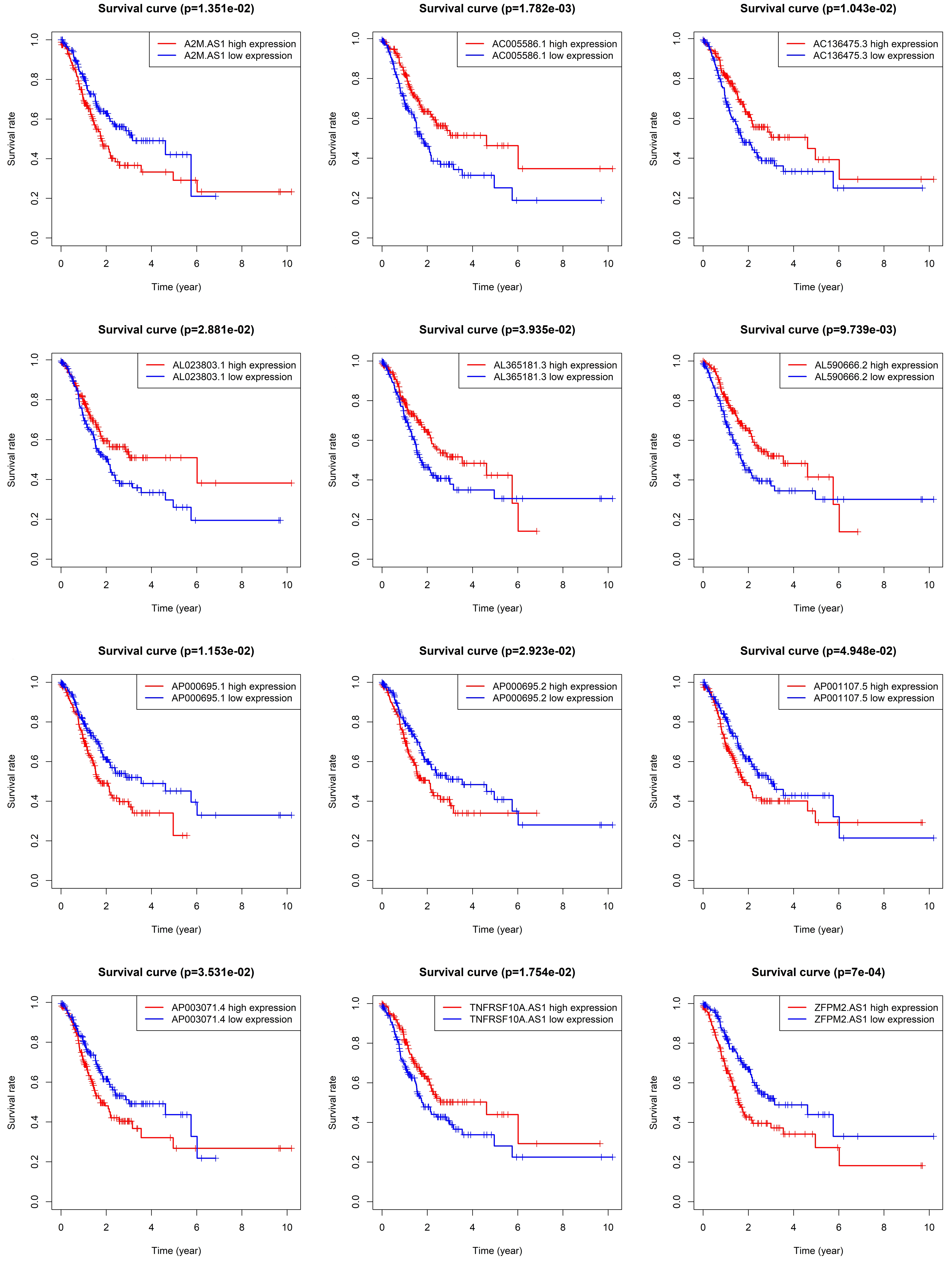

Supplementary Figure 3

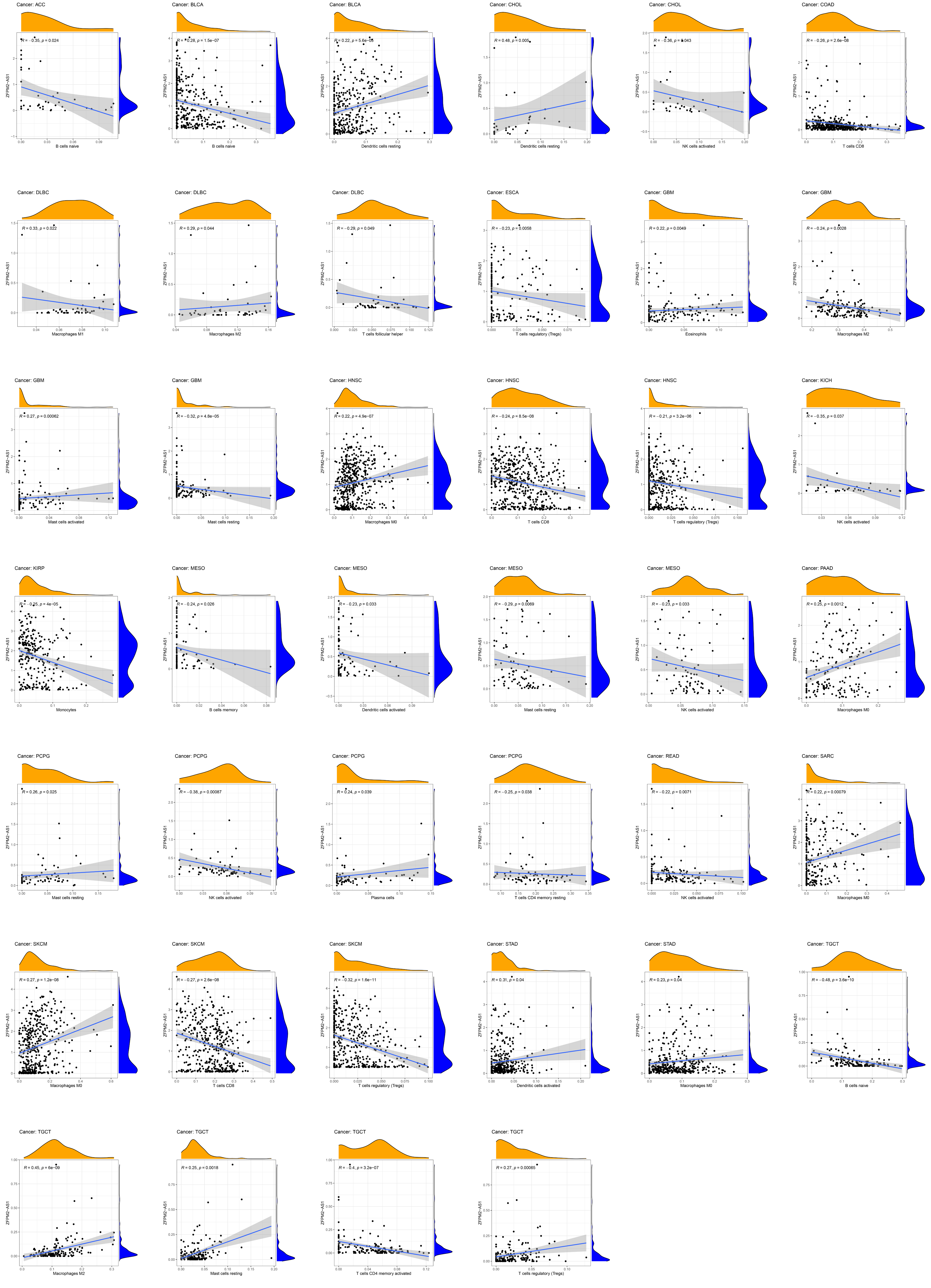

Supplementary Figure 4

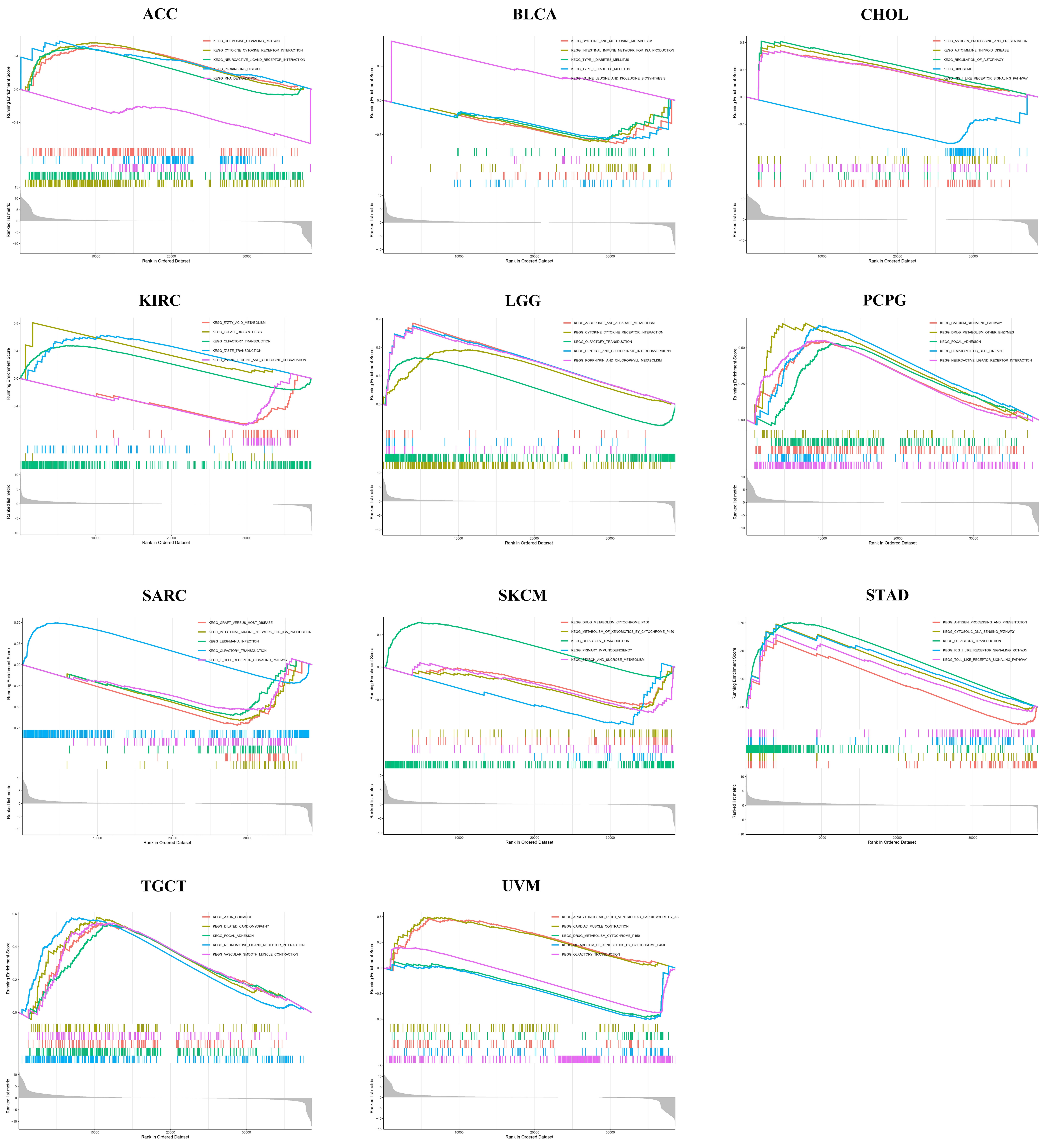

Supplementary Figure 5

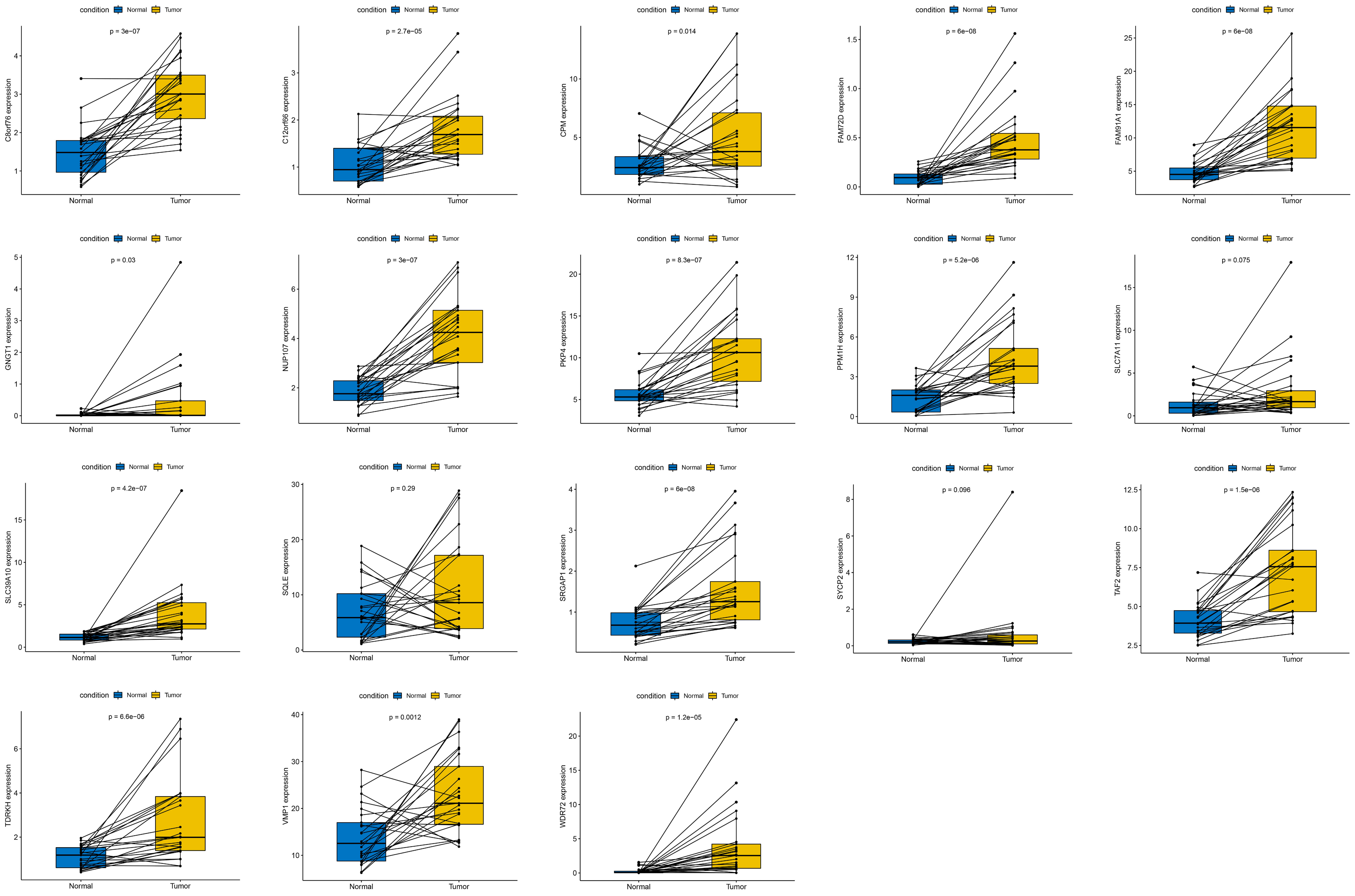

Supplementary Figure 6

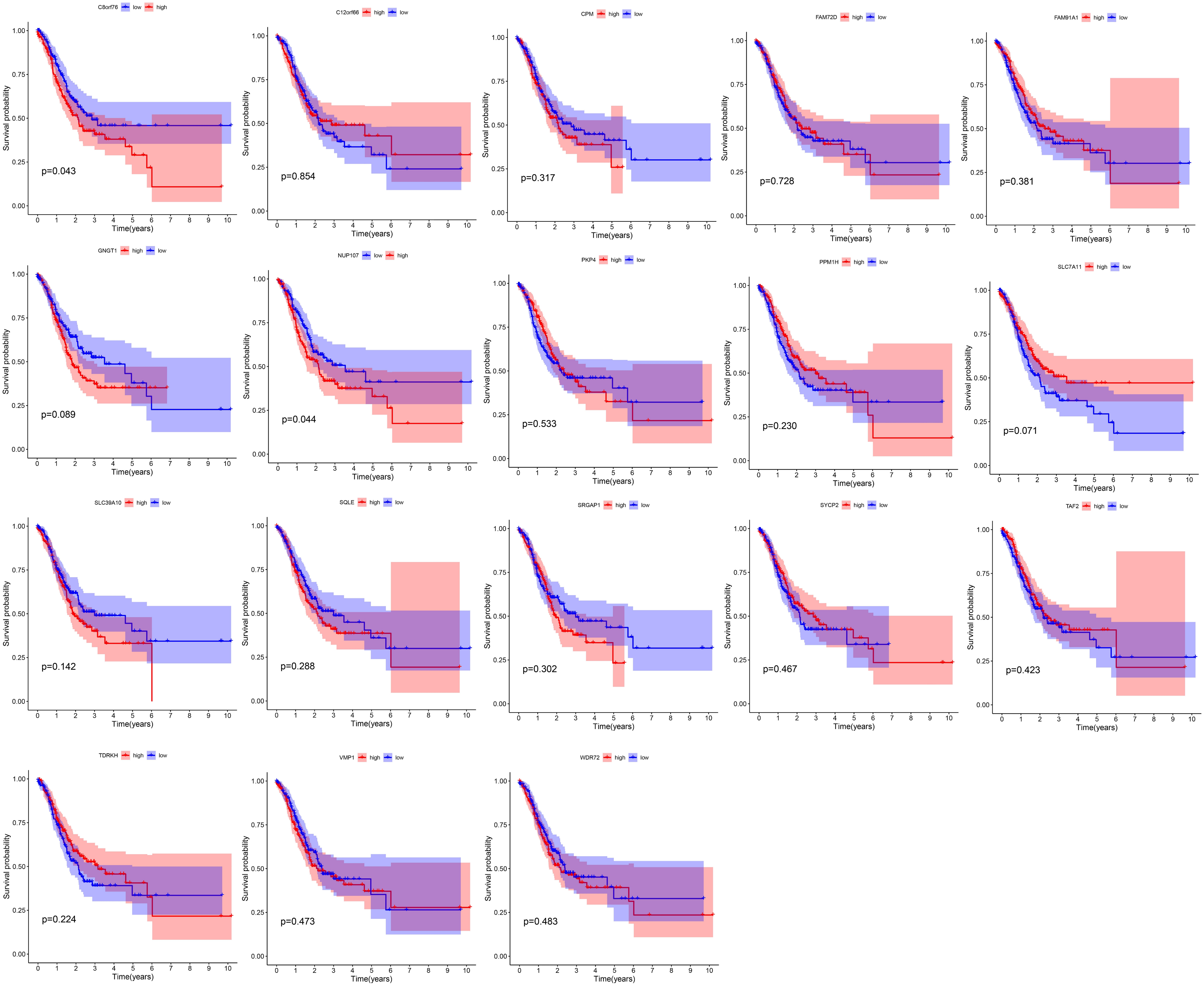

Supplement: Supplementary file 1 — (PDF 21189 KB) [file 12672_2022_487_MOESM1_ESM.pdf]
